# Ecology and Genetics of Natural Populations of North American *Vitis* Species Used as Rootstocks in European Grapevine Breeding Programs

**DOI:** 10.3389/fpls.2020.00866

**Published:** 2020-06-19

**Authors:** Claire Arnold, Annik Schnitzler

**Affiliations:** ^1^ Unicentre, University of Lausanne, Lausanne, Switzerland; ^2^ LIEC - UMR 7360 - CNRS, University of Lorraine, Metz, France

**Keywords:** *Vitis*, Vitaceae, ferality, floodplain, populations ecology and genetics, Arkansas, rootstock, invasion risk

## Abstract

Three North American *Vitis* species (*V. riparia, V. berlandieri, V. rupestris*) became widely used in rootstock breeding programs following the expansion of North American pests and diseases introduced in vineyards of the world during the 19th century. When they escape, they become feral in the most dynamic parts of Mediterranean floodplains. To better understand this ongoing process, we studied the ecology of Vitis species in their native sympatric range. We analyzed in deep 61 plots of 710 m2 containing Vitaceae species along 216 km of the Buffalo River and adjacent plateaus (Arkansas, United States). We investigated the populations structure and genetics of the *Vitis* complex (i.e., possible hybrids and the *Vitis* species) and the sharing of habitats with other Vitaceae (*Muscadinia rotundifolia* and *Parthenocissus quinquefolia*). Vitaceae share space according to their life strategies and microhabitat along ecological gradients. The plateau niche seems optimal for *V. berlandieri* and *V. aestivalis*. *V. berlandieri* is also found in alluvial zones. The most erosive parts of the river are colonized by *V. rupestris*, whereas the first terraces include most of the *M. rotundifolia* populations. *Vitis riparia* and *Parthenocissus* live in the largest range of forest habitats, from plateaus to alluvial forests, and from the forest floor to the canopy, with the highest densities along the river. Interestingly, natural hybridization can occur, but establishment success is rare and limited to alluvial forests. In their native range, these populations are controlled by biotic and abiotic conditions. In Europe, the biotic relations among species are different. Our study shows that *V. riparia* and its hybrids could be the best candidates for a large scale invasion.

## Introduction

### American *Vitis* Species

In the 19th century, artificial breeding between North American *Vitis* species was necessary to produce rootstocks and grape varieties resistant to American pests and diseases. *Vitis riparia* Michx., *V. berlandieri* Planch (syn. *V. cinerea* var. *helleri* (Bailey) M.O. Moore), and *V. rupestris* Scheele were used as rootstocks for most of the cross-breeding and breeding programs in Europe and the New World (Pongracz, 1983; Reynolds, 2015 by Morano and Walker, 1995; Legros, 2005 *)*. Other American species like *V. aestivalis* Michx were used extensively in rootstock breeding (Einset and Pratt, 1975).

American *Vitis* species are dioecious and interfertile (Heinitz et al., 2019). *V. riparia* and *V. rupestris* are known to root easily from woody cuttings and to graft well with *Vitis vinifera* (Riaz et al., 2019). It is thus easy to reproduce them vegetatively and to graft grape varieties on them. *V. berlandieri* is indigenous to the calcareous mountains of central Texas and was introduced because most European vineyards are cultivated on similar soils. Other rootstocks suffer from lime-induced iron chlorosis. The beneficial traits of *V. aestivalis* are, among others, high vigor, disease resistance and environmental stress tolerance (Mortensen et al., 1990).

Rootstocks were collected by American plant breeders or during various field trips in the United States at the end of the 19th century (Viala, 1889). For instance, the rootstocks Kober 5BB and SO4 are hybrids of *V. berlandieri* x *V. riparia* (Riaz et al., 2019). 99 Richter is a crossing between *V. berlandieri* and *V. rupestris,* and Couderc 3309 resulted from cross-breeding between *V. riparia* and *V. rupestris* (PlantGrape 2009–2011). Most of these rootstocks were named after famous breeders of the time, such as Teleki, Rességuier, Kober, Paulsen, and Ruggeri (Ruehl et al., 2015), and they were bred to match vineyard conditions (PlantGrape, 2009-2011; Heinitz et al., 2019).

Many of these artificial hybrids used as rootstocks escaped from the vineyards at least in Europe, either after the abandonment of viticulture, or locally from shoots left around cultures. Generally, escaped rootstocks establish close to vineyards, where female individuals produce fruits that attract dispersers. Adult plants are usually observed along rivers, roads, dykes, and fallows (Laguna, 2003; Arrigo and Arnold, 2007; Arnold and Schnitzler, 2008; Zecca et al., 2009; Melendez et al., 2016; Arnold et al., 2017). The survival of rootstocks can principally be explained by their tolerance to downy, powdery mildew, and phylloxera (Laguna, 2003; Melendez et al., 2016). Also, they seem to have good uptake of nutrients and water in arid zones, as shown in grafted cultivars whose growth is enhanced compared with ungrafted cultivars (Candolfi-Vasconcelos et al., 1997; Toumi et al., 2007; Medrano et al., 2015; Uretsky, 2018). Third, it is likely that with time, colonizing rootstocks became feral, i.e., they came to differ from their parent populations due to the emergence of novel traits better adapted to local environmental stresses, a process described by Gressel (2005) for crop species, including grapevines. All these factors enhance the adaptability of escaped rootstocks for establishing in anthropogenic habitats (Concept of invasiveness Pyšek and Richardson, 2008), in particular along Mediterranean rivers characterized by high dynamics and sandy, calcareous substrates. Alluvial forests are here deciduous and naturally liana-rich forests (Arnold and Schnitzler, 2008).

These feral populations are present in habitats characteristic of the Eurasian *Vitis vinifera* ssp *sylvestris* (screes or alluvial forests) but have also colonized new habitats such as hedges, streamside hedges (Arrigo and Arnold, 2007), and the most erosive parts of floodplains (Arnold et al., 2017). As a result, current wild populations of *Vitis* are made up of interspecific individuals with enormous genetic diversity and benefiting from exchanges with several genes of resistance to biotic or abiotic stresses (Galet, 1989; Laguna, 2003; Arrigo and Arnold, 2007). Moreover, natural interspecific crossings between feral *Vitis* and the native grapevine *V. vinifera* have been detected, and these have led to the emergence of a genetic complex of wild forms, rootstocks, naturalized domesticated forms and hybrids derived from spontaneous hybridizations and introgressions among these taxa (Levadoux, 1956; Lacombe et al., 2003; Laguna, 2003; Laguna, 2004; Warwick and Stewart, 2005; Lowe and Walker, 2006; This et al., 2006; Arrigo and Arnold, 2007; Arnold and Schnitzler, 2008; Zecca et al., 2009; Bodor et al., 2010; Ocete et al., 2011). This process has contributed to the eradication of the endemic wild grapevine *V. vinifera* ssp *sylvestris*, already endangered by American pests and pathogens and large-scale habitat destruction (Arnold et al., 1998; Arnold et al., 2010). Only a few sites are still preserved against the invasion of exotic *Vitis* (Arnold, 2002, Arnold et al., 2005; Arnold et al., 2010; Arnold et al., 2017; Schnitzler et al., 2018).

The scarcity of the wild grapevine in comparison to the expansion of feral hybrid taxa points to the need to better understand the ecology of *Vitis* species used as rootstocks and their invasive potentialities through the emergence of new variants. Yet very few studies have been carried out on *Vitis* ecology in North America. In 1889, Viala noted that better knowledge of the environment in which American vines grow could have prevented, or at least predicted, the failures of the first rootstock programs. Almost all published studies have focused on aspects related to agronomy (genetics, phenology, stresses, soils) and industry (natural compounds) (Heinitz et al., 2019). For example, Grando et al. (2003); Lowe and Walker (2006) and Pap et al. (2015) have compared genomes between the Eurasian *Vitis* and other *Vitis* species in order to study the resistance and productivity traits in grape varieties. Xiao et al. (2008) focused on the genes implicated in the adaptation of *V. riparia* to freezing and drought, while other researchers have evaluated the *Vitis* resistance to pests and diseases (Peros et al., 2015; Smith et al., 2018).

In contrast, the ecological aspects of natural settings have been systematically trivialized. Researchers in viticulture have collected plants in natural settings but neglected to report on the type of environment or the behavior of these species in their natural environment (Pap et al., 2015). Although today the combined knowledge of ecological and evolutionary processes is important for developing more sustainable agriculture, these studies remain rare in the domain of crop-related species—and this despite the high probability that many problems in agriculture and plant invasion can be avoided through a better preliminary understanding of the ecology and genetics of species within their native range. Unfortunately, such approaches tend to develop only when crops or horticultural plants become feral and have a negative economic impact.

The exception is the biogeographical and ecological work carried out in the southern part of the US by Morano and Walker (1995) on three species: *V riparia, V. berlandieri,* and *V. rupestris.*

In this study, the authors aimed to determine their composition and the environmental characteristics of their habitats in 24 riparian sites at the scale of central Texas, eastern Oklahoma, southeastern Kansas, southern Missouri and western Arkansas, all of which include them (Smith, 1988) see also USDA plant data base (USDA, 2019). Results indicate that their habitats were distinct: *V. berlandieri* had the greatest success on dry and limestone soils; *V. riparia*, in sandy moist soils; and *V. rupestris*, in moist and gravelly soils.

For the current study, we aimed to better understand the dynamic expansion of interspecific feral *Vitis* rootstocks, especially in the Mediterranean climatic conditions. We selected a region in the southern part of North America that is relatively similar to the most invaded Mediterranean zones in Europe. Within this region, we selected the alluvial valley of the Buffalo River and the adjacent upland deciduous forests, because there, the *Vitis* species used in rootstock breeding programs coexist. Data were collected in Vegbank and American vegetation surveys, including that of Schnitzler et al. (2016).

Our objective was to (i) clarify the sharing of space among *Vitis* species (and other Vitaceae) in their native range, and (ii) look for potential *Vitis* hybrids and evaluate their specific life strategies in a sympatric region. On the basis of our results, we discuss possible scenarios for the evolutionary history of the native Eurasian grapevine following the increasing presence of alien *Vitis.*

### Study Area

**Figure 1:**
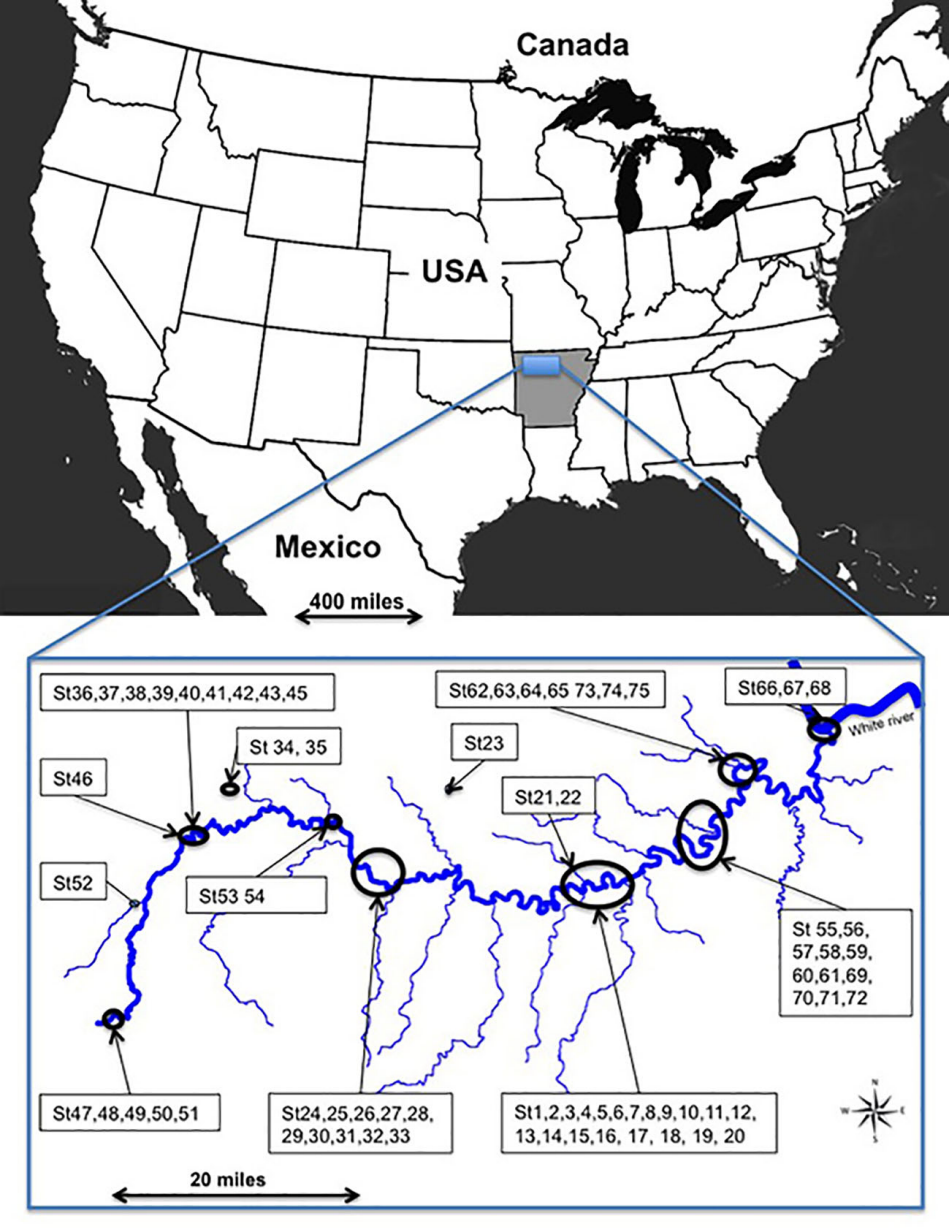
Study site along the Buffalo River and its main tributaries in Arkansas, United States.

The study site (92°44' to 93°46' N; 35°97' to 36°16'E) encompassed 347,068 ha in Arkansas, at altitudes from 117 to 340 m along the river, and 660 m on upland plateaus (**Figure 1**). The climate is warm temperate continental. Mean temperature is 16°C with long hot summers and mild winters; mean annual rainfall of 1206 mm. Rainy periods occur mainly in winter.

The Buffalo River has cut downwards but has also eroded the three Ozark plateaus laterally (Boston Mountains, Springfield and Salem plateaus) and flows 241.5 km to the east up to its confluence with the White River. Vertical ledges and cliffs of hard sandstone and limestone alternate with sloping terraces. A large part of the Buffalo River's watershed is surfaced with carbonate rock. Along the river, erosion and sedimentation constantly rework the landscape, which explains the complex sedimentological pattern with temporary islands, terraces, hollows, backwater depressions, wetlands, secondary arms, and old channels periodically connected with the river (**Supplementary Material, SM1**).

Forests are deciduous and belong to several groups. Bottomland hardwood forests are flooded at least once a year; they include American elm (*Ulmus americana* Brandon), box elder (*Acer negundo* L.), green ash (*Fraxinus pennsylvanica* Marshall), and sweet gum (*Liquidambar styraciflua* L.). Stream terraces along the river stand a bit higher in elevation; woods include most of the floodplain trees with also black walnut (*Juglans nigra* L.), blue beech (*Carpinus caroliniana Walter*), and iron wood (*Ostrya virginiana* (Mill.) K. Koch). These rich, moist woodlands have displays of wildflowers blooming in early spring before trees leaf out. Mixed hardwoods are found in moist ravines. Oak-hickory (*Quercus-Carya* sp) woodlands cover the upper terraces and the plateaus; oak-pine (*Quercus-Pinus*) woodlands occur in patches on acid soils. There exist also post-successional pine forest and post-successional oak forests.

Forests are liana-rich, except for the drier ones such as the pine forests of the plateaus. We observed four Vitaceae genera: *Vitis (V. riparia, V. rupestris, V. berlandieri, V. aestivalis); Ampelopsis (A.arborea* (L.) Koehne and *A. cordata* Michx*.); Muscadinia rotundifolia* Michx*. (*previously named *Vitis rotundifolia) and Parthenocissus quinquefolia* (L.) Planc. The other liana are *Bignonia capreolata* L***.;*** *Berchemia scandens* (Hill) K. Koch*; Brunnichia ovata* (Walter) Shinners*; Dioscorea (D. quaternata* J.F.Geml*., D. oppositifolia* L*.); Campsis radicans* (L.) Seem.*; Toxicodendron radicans* (L.) Kuntze (Tucker, 1976; Smith, 1988; Moore, 1991; Hunter, 2000).

The landscape had been heavily exploited for timbering, zinc mining, clearing for agriculture, and trapping for 200 years. Roads were created, and riverbanks were deforested and stabilized. In 1972, however, 8 years after the Wilderness Act (1964), the Buffalo National River Act was signed, mandating its reversion to nature, and most of the river and its shorelands were left to free evolution. In 1975, Congress created the Upper Buffalo Wilderness in the Ozark National Forest and later designated three wilderness areas within the National River range. The present size of the National Forest Upper Buffalo Wilderness is 11.094 acres (45 km^2^). After 50 years of free development, forests have partially recovered their potential areas, but they still bear evidence of human alteration (Smith, 2004).

Since the establishment of protective laws, at least three big floods have occurred. We started the project in the Buffalo River region in May 2011 at the end of heavy flooding. Some islands had moved and most of the vegetation on the first terraces had been washed out or was covered by sand.

## Methods

Seventy-five sites containing *Vitis* species were investigated in May 2011 along the 216 km protected zone of the Buffalo River, including the floodplain and the above plateaus (**Figure 1**). Given the heterogeneity of the landscape, study of small plots is more appropriate. Thus, in each site, we took a tree as a central point and drew a circular plot of 710 m² around the tree with a 15 m rope. *Vitis* species were identified according to morphological characters based on determination keys (Tucker, 1976; Smith, 1988; Everhart, 2010). When leaves or young shoots were accessible, we collected them either to confirm the morphological identification by genetic analysis or the suspected presence of hybrids and their pedigree.

We also collected leaves of *Muscadinia rotundifolia*, because this plant was until recently included in the *Vitis* genus. It does not have the same chromosome number, but cross-breedings have been made in viticulture (Alleweldt et al., 1991; Lu et al., 2000; Conner, 2006), which suggests that it might occur in nature (Alix et al., 2017).

### Genetic Analysis

Leaves or shoots of *Vitis/Muscadinia* samples were dried in silica gel and stored at -20°C until extraction. Genomic DNA was extracted with the DNeasy Plant Mini Kit (Qiagen), according to the manufacturer's instructions. Twenty-three SSR primers were used; all were developed on *Vitis* species (**Table 1**). The primers had already been tested on accessions of *Vitis* species and common rootstock clones from the collections of the Institut für Rebenzüchtung Geilweilerhof (Germany) and from the Agroscope Viticulture Research Center Pully (Switzerland), as well as on populations of escaped rootstocks (Arrigo and Arnold, 2007; Arnold et al., 2017; unpublished studies O. Bachmann).

**Table 1:** List of the 23 SSP primers, annealing temperature, number of alleles, missing data in the five species in percentage and references.

SSR Primers	Annealing Temperature	Total No. of alleles	Missing data *M. rotundifolia* %	Missing data *V. aestivalis* %	Missing data *V. berlandieri* %	Missing data *V. riparia* %	Missing data *V. rupestris* %	References
**VMC 2F10**	56	16	11	11	0	0	0	Vitis Microsatellite Consortium
**VMC 5G8**	56	22	0	33	33	6	0	Vitis Microsatellite Consortium
**VMC 6E10**	55	14	100	22	6	0	0	Vitis Microsatellite Consortium
**VMC 7F2**	56	12	16	22	0	0	0	Vitis Microsatellite Consortium
**VMC 8G6**	54	19	37	11	50	26	40	Vitis Microsatellite Consortium
**VMC 9B5**	56	19	0	33	11	2	0	Vitis Microsatellite Consortium
**ZAG 112**	56	30	0	22	11	0	0	Sefc et al., 1999
**ZAG 83**	56	14	5	33	11	0	0	Sefc et al., 1999
**VMC 1E8**	56	28	95	56	11	16	0	Vitis Microsatellite Consortium
**VMC 2A5**	56	23	0	33	33	10	0	Vitis Microsatellite Consortium
**VMC 5A1**	56	21	0	0	0	0	0	Vitis Microsatellite Consortium
**VMC 5C5**	56	11	47	33	11	4	0	Vitis Microsatellite Consortium
**VVMD 24**	56	15	0	11	0	0	0	Bowers et al., 1999
**VVMD 25**	56	20	89	11	11	6	0	Bowers et al., 1999
**VVMD 31**	53	19	42	22	6	10	0	Bowers et al., 1999
**VVMD 32**	56	32	74	22	11	6	0	Bowers et al., 1999
**VMC 2B11**	56	6	5	22	11	4	0	Vitis Microsatellite Consortium
**VMC 3D12**	56	16	89	33	39	10	0	Vitis Microsatellite Consortium
**VMC 4G6**	56	15	0	56	28	28	0	Vitis Microsatellite Consortium
**VVMD 7**	52	10	63	11	17	8	0	Bowers et al., 1996
**VVS 2**	52	26	100	22	0	0	0	Thomas and Scott, 1993
**ZAG 62**	56	26	5	11	22	0	0	Sefc et al., 1999
**ZAG 79**	56	20	21	11	11	2	0	Sefc et al., 1999

Amplifications were carried out in 10 µl reactions containing 1x GoTaq Reaction Buffer, 0.75 mM MgCl2, 5µg BSA, 0.25 mM dNTPs, 0.25 µM of each primer, 0.5 U GoTaqG2 DNA Polymerase (Promega), and 2-5 ng of template DNA. The PCR cycling conditions consisted of an initial activation step of 4 min at 94°C, followed by 30 cycles each of 60 s at 92°C, 50 s at 52-56°C (**Table 1**), and 60 s at 72°C, with a final extension step of 10 min at 72°C.

Genotyping was performed by the service provider Macrogen Europe BV (the Netherlands).

Amplified fragment lengths were assigned to allele sizes with GeneMapper software v 3.7 (Applied Biosystems).

We carried out a STRUCTURE 2.3.4 analysis on the individuals that amplified (Pritchard et al., 2000). The goal was to confirm the morphological identification of the five *Vitis* species (including *Muscadinia rotundifolia*, formerly *Vitis rotundifolia*) identified in nature and to determine whether hybridization or introgression had occurred along the Buffalo River. The following options were used: 10,000 burn-in, 100,000 Markov chain Monte Carlo (MCMC) simulations, an admixture model and correlated allele frequencies. This method is based on the use of MCMC simulations to infer the assignment of genotypes to distinct K-means clusters. The underlying algorithms attempt to minimize the deviations from the Hardy-Weinberg Equilibrium and linkage disequilibria within each cluster. In accordance with Evanno et al. (2005), we performed 10 iterations for each K value (K=1 to K=6). We focused on K=5 corresponding to the five *Vitis* species. For the identification of hybrids, the threshold was set at 50%, and for the identification of introgressed individuals, the threshold was set at 20%. However, we went back to the raw data to validate these results.

Frequency-based statistics were calculated in GenAlEx 6.5. (Smouse and Peakall, 2012): number of different alleles (Na), number of effective alleles (Ne), Shannon's Information Index (I), observed heterozygosity (Ho), expected heterozygosity (He), percentage of polymorphic loci (%P) and number of private alleles (PAL) (**Table 2**).

**Table 2:** Summary of genetic diversity in the five Vitaceae species present along the Buffalo River.

Pop		Na	Ne	Ho	He	I	%P	PAL
***V. aestivalis***	**Mean**	5.739	4.390	0.740	0.726	1.515	100.00%	23
	**SE**	0.437	0.341	0.060	0.030	0.092		
***V. cinerea /***	**Mean**	6.261	3.963	0.570	0.610	1.330	95.65%	40
***V.berlandieri***	**SE**	0.709	0.517	0.058	0.057	0.148		
***M. rotundifolia***	**Mean**	2.957	2.145	0.349	0.352	0.673	60.87%	28
	**SE**	0.489	0.334	0.080	0.070	0.140		
***V. riparia***	**Mean**	13.957	6.696	0.687	0.762	1.992	100.00%	136
	**SE**	1.143	0.836	0.046	0.040	0.140		
***V. rupestris***	**Mean**	3.739	3.213	0.713	0.674	1.213	100.00%	10
	**SE**	0.144	0.142	0.063	0.016	0.044		

Na, number of alleles; Ne, effective number of alleles; Ho and He, observed and expected heterozygosities, respectively; I, Shannon's Information Index; %P, percentage of polymorphic loci; PAL, number of private alleles per species.

### Ecological Analysis

For this part of the research, we considered all Vitaceae (*Parthenocissus quinquefolia*, *Vitis* sp. and *M. rotundifolia).* They have common life strategies and interfere probably more between them than with other lianas. Two analyses were performed:

1. An analysis of shared space along the river and within the forest strata, performed at the level of the Vitaceae genera (*Vitis; Muscadinia; Parthenocissus*). For this purpose, we counted the number of these species in the canopy and the bush strata (i.e., 2–7 m). We also measured the heights and diameters (so-called DBH=diameter at breast height) of the highest lianas to evaluate their prevalence in the canopy. Heights were measured with a Bosch PLR 30 digital laser (in meters) or by biometric techniques. Diameters were measured perpendicularly with a caliper gauge.
   We compared the number of *Vitis*, *Muscadinia,* and *Parthenocissus* in the canopy and the bush layer using a Kruskal test, as the data failed to met normality of distribution and homoscedasticity criterions. The Kruskal test and Wilcoxon rank test were applied for diameters in the canopy, as normality of distribution and homoscedasticity criteria were also not met.
  The coverage of Vitaceae in the herbaceous strata (i.e., 10 to 100 cm, corresponding to both sexual and asexual plants) was estimated using semi-quantitative values issued from the phytosociological analysis of vegetation (estimation of plant coverage from 1 to 5 with 1: <10%; 2: 10% to 25%; 3: >25%–50%; 4: >50% to 75%; 5: >75% to 100%) (Oberdorfer, 1992).
2. A detrended correspondence analysis (DCA) was performed with the use of Canoco 4.5 software (Lepš and Šmilauer, 2003) at the “*Vitis”* species level (*V.riparia, V. aestivalis, V. rupestris, V. berlandieri, M. rotundifolia* and *Vitis* hybrids). We built up a matrix, regrouping the sites in rows and the six species variables plus the 21 ecological variables in columns. The six species variables were recorded by presence or absence in the station. The 27 variables are shown in **Table 3**. Among these variables, we included the mean height of the canopy and the under-canopy, as well as the number of trees according to their maximum diameters (>30 cm, between 10 cm and 30 cm, and <10 cm). We have considered separately the first and the second alluvial terraces. The first terrace, closer to the river and of low elevation, is more regularly submitted to floods.
  A detrending method by second-order polynomials was used. We did a square root transformation of the data and a downweighting of rare species. We removed five highly correlated variables (UndCa, D10-30, NMusC, Dim10, NParC) and ran a second DCA.
  We created a biplot diagram in Canodraw and interpreted the projection of the first two axes of the DCA.

**Table 3:** Types of descriptors.

	Abreviation	Description	Type of data
1	*V. riparia*	Presence of the species in the plot	binary
2	*V. hybrids*	Presence of hybrids or introgressed individuals in the plot	binary
3	*V. aestivalis*	Presence of the species in the plot	binary
4	*V. rupestris*	Presence of the species in the plot	binary
5	*V. cinerea /* *V. berlandieri*	Presence of the species in the plot	binary
6	*M. rotundifolia*	Presence of the species in the plot	binary
1	Dip30	Number of trees with a diameter larger than 30 cm	quantitative
2	D1030	Number of trees with a diameter between 10 and 30 cm	quantitative
3	Dim10	Number of trees with a diameter smaller than 10 cm	quantitative
4	Islnd	Plot located on an island	binary
5	RipFo	Plot located in a riparian forest	binary
6	Tce1	Plot located on the first alluvial terrace	binary
7	Tce2	Plot located on the second alluvial terrace	binary
8	NonAl	Plot located outside the influence of flooding	binary
9	C.O.	Canopy openess in percentage	quantitative
10	CanoH	Height of the canopy in meters	quantitative
11	UndCa	Height of the undercanopy in meters	quantitative
12	NVitC	Number of *Vitis* in the canopy	quantitative
13	DmaVC	Maximum diameter of the *Vitis*	quantitative
14	NVitB	Number of *Vitis* in the bush strata (2 to 7 m)	quantitative
15	NMusC	Number of *Muscadinia* in the canopy	quantitative
16	DmaMC	Maximum diameter of *Muscadinia* in cm	quantitative
17	NParC	Number of *Parthenocissus* in the canopy	quantitative
18	DmaPC	Maximum diameter of *Parthenocissus* in cm	quantitative
19	NParB	Number of *Parthenocissus* in the bush strata (2 to 7m)	quantitative
20	PartH	Ground covered by *Parthenocissus* up to 50 cm	semi-quantitative
21	VitiH	Ground covered by *Vitis* up to 50 cm	semi-quantitative

## Results

### Sample Numbers for Each Analysis

For the 75 plots, we have data on the presence of the different genera and ecological data. In five plots, it was not possible to reach leaves or shoots. In the 70 remaining stations, we collected between one and three individuals of different species or suspected hybrids. In total, we collected 113 samples (leaves or shoots). In nine plots where we collected only one individual, we had no amplification (70-9 = 61 stations) (113-9 = 104 individuals). In station 9, the *M. rotundifolia* did not amplify. In station 30, the *V. riparia* did not amplify, and in station 50, the *V. riparia* again did not amplify (104-3 = 101). In summary, we had a total of 101 genetically identified individuals in 61 stations homogeneously distributed along the Buffalo River. The first four signs indicate the station number; the next indicates the sample number within the station (ST01-x to ST75-x).

### Genetics of Vitaceae Genera

23 SSR primers were amplified in *Vitis* (**Table 1**). The sizes were within the expected ranges (**Supplementary Material SM3**). However, the success depended on the species. *M. rotundifolia* had a high rate of missing data for several primers. VMC 6E10 and VVS 2 did not amplify at all, VMC 1E8 had 95% missing data, whereas VVMD 25 and VMC 3D12 had 89% missing data. In *Vitis riparia*, V *berlandieri*, V. *rupestris,* the missing data for the primers reached a maximum of 50% for VMC 8G6 in *V. berlandieri*. All primers were polymorphic, and VVMD 32 showed the highest number of alleles (32). VMC 2B11 had only six different alleles.

**Figure 2:**
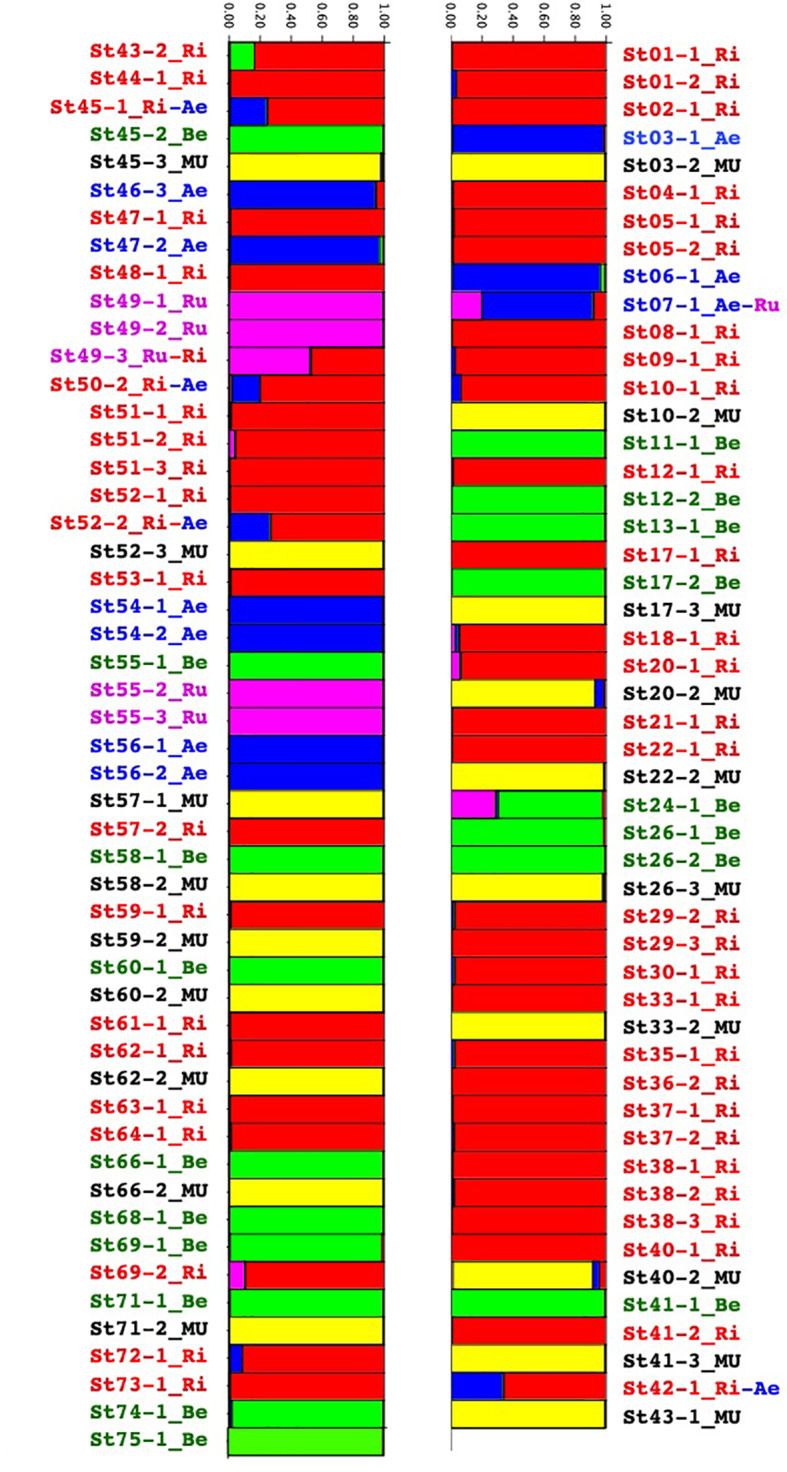
Group assignment through Bayesian clustering analysis with prior population assignment of the *Vitis* species of the Buffalo River using STRUCTURE population genetics software.

In the structure analysis (**Figure 2**), we only considered K=5 where the five species could be confirmed.

*M. rotundifolia* was present in 28 of the 61 sites but we collected only 19 of them for genetic analysis. *V. riparia* was present in 42 sites, and *V. berlandieri* was found in 17 sites. These two species were present from the source to the river mouth. *V. aestivalis* was along the whole river in seven sites. *V. rupestris* was only present in two sites, one close to the source of the Buffalo River and the other 50 km from Buffalo City where the river flowed into the White River (**Supplementary Material SM2** and **SM3**).

Eight individuals seemed to be either hybrids or introgressed (**Figure 2**). A closer look at the raw data nevertheless suggested that the ST24-1 and ST43-2 samples had many loci that did not amplify. Regarding the six left: ST07-1, ST42-1, ST45-1, ST49-3, ST50-2, and ST52-2, we suspected natural hybridization and introgression through insect vectors (mostly bees) between *V. aestivalis, V.rupestris,* and *V.riparia.* In this sympatric region, they were all located close to the river, except for ST07-1 located on the plateau.

In the five species, loci were polymorphic. Species presented large numbers of private alleles (**Table 2**). For example, there were 136 private alleles present in *V. riparia*. The observed and expected heterozygosities were relatively similar between the *Vitis* species and also high: between 0.570 and 0.762. *M. rotundifolia* showed a lower genetic diversity with Ho at 0.349 and He at 0.352.

### The Prevalence of the Three Vitaceae Genera With Regard to Forest Stratification

**Table 4** shows that the result is significant both for the canopy and the bush (p<0.001). With an epsilon squared = 0.136, effect size is considered as medium (Vargha and Delaney, 2000). Pairwise comparison using Wilcoxon rank test shows that in both cases, *Muscadinia* is different from both *Parthenocissus* and *Vitis* (p<0.001), whereas *Parthenocissus* and *Vitis* are non-significantly different (p=0.32).

**Table 4:** Results for the vine in regard to diameter (DBH) and number (N) in the canopy and in the bush strata (median [range]).

Genera	DHB (cm)	N (canopy)	N (bush)
*Vitis*	6.5 [23]b	4 [15]a	0 [50]a
*Parthenocissus*	2 [4]a,c	3 [16]a	0 [7]a
*Muscadinia*	6 [10]b	0 [10]b,c	0 [2]b,c

Significant difference with a: Muscadinia, b: Parthenocissus, and c: Vitis.

**Table 4** also shows that diameter differs significantly as a function of the genus (p<0.001). The effect size can be considered as large (epsilon squared = 0.482). Pairwise comparison using Wilcoxon rank test shows that the diameter of *Parthenocissus* differs significantly from *Muscadinia* (p<0.001), *Parthenocissus* differs also significantly from *Vitis* (p<0.001) while *Muscadinia* and *Vitis* are not significantly different.

Vitaceae in the herbaceous strata were present in 20% and 56% of the total number of sites for *Vitis* and *Parthenocissus*, respectively. *Muscadinia* was absent. Generally, they did not live in sympatry (only 15 sites include both species). Coverage of seedlings was low (<10% to <25%).

### Comparative Ecology of the *Vitis* Species

The total variance for the data (inertia) in the DCA was 0.566. The first four canonical axes explained 59.3% of the total variance (Axis 1 = 26%, Axis 2 = 18.1%, Axis 3 = 8.6%).

**Figure 3:**
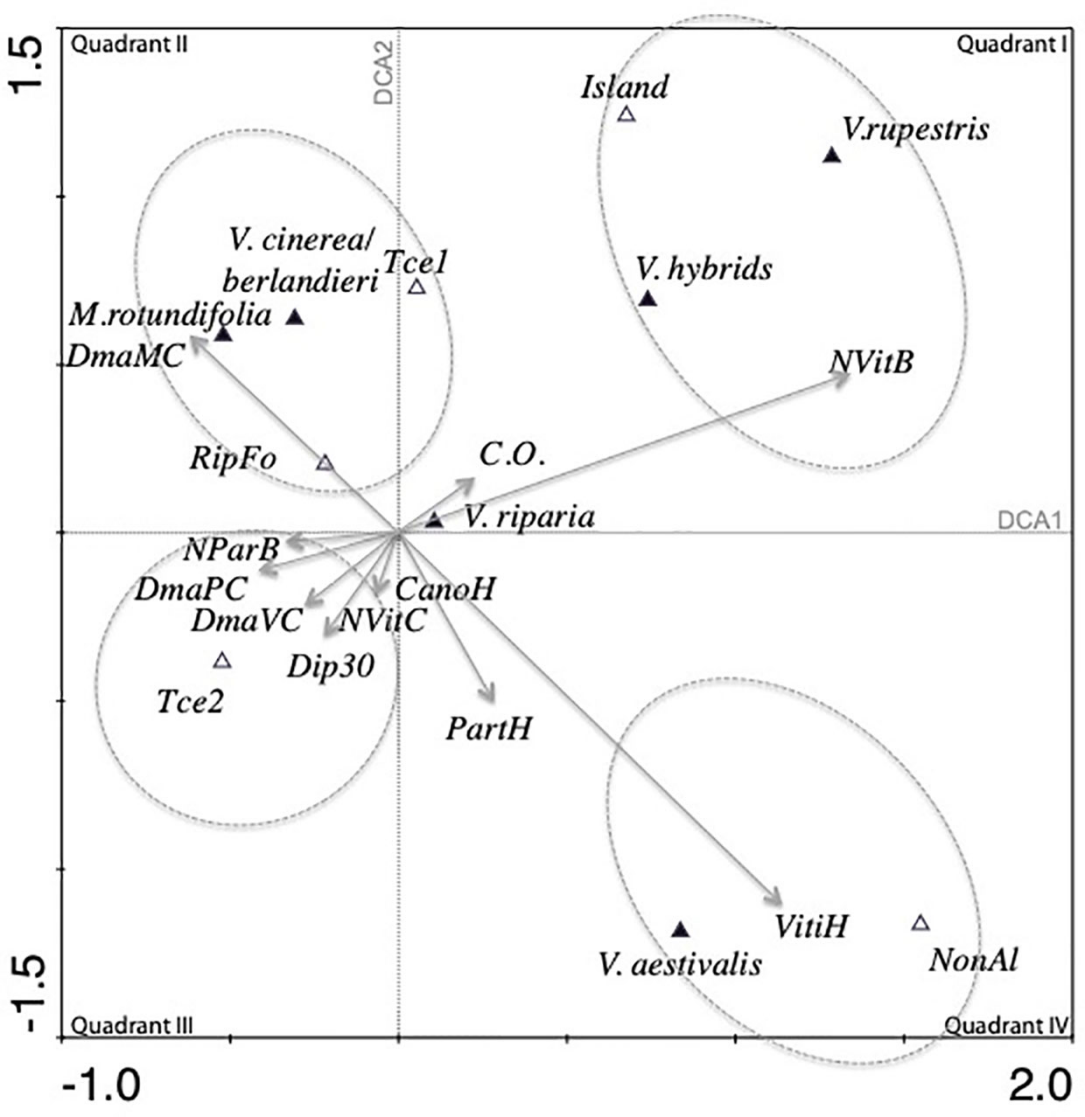
Projection of the species and environmental variables on the first two axes of the detrended correspondence analysis (DCA) (61 plots and a selection of 22 variables). Quantitative and semi-quantitative variables are presented by arrows, and binomial variables are presented by their centroids.

In the first quadrant of the simple ordination plot of the DCA (**Figure 3**), the number of *Vitis* in the bush strata (NVitB) was correlated with the opening of the canopy (C.O.). *V. rupestris* was predicted to have its optimum with respect to the number of *Vitis* in the bush strata (NVitB), canopy openness (C.O.), and location on islands; *Vitis* hybrids and *V. rupestris* shared similar ecology.

*V. cinerea/berlandieri*, *M.rotundifolia*, presence on the first terrace (Tce 1) and presence of riparian forest (RipFo) formed a cluster. *M. rotundifolia* and *V. cinerea* were linked to the large diameter of *M. rotundifolia* in the canopy (DmaMC). In quadrant III, another cluster regrouped the number of *P. quinquefolia* in the bush strata (NParB), maximum diameter of *P. quinquefolia* (DmaPC) and *Vitis* (DmaVC) in the canopy, large trees (Dip30), canopy height (CanoH) and presence in the less dynamic second alluvial terrace (Tce2). In quadrant IV, estimation of the ground covered by *Vitis* (VitiH) was strongly correlated with estimation of the ground covered by *P. quinquefolia* (PartH). Presence of *V. aestivalis* and presence of the plot in a non-alluvial environment (NonAl) had the largest average value of variable PartH and VitiH. C.O. and abundance of *Vitis* in the herbaceous strata (VitiH) were independent and were negatively correlated with the maximum diameter of *Vitis* in the canopy (DmaVC). *V. riparia* had a central position in the simple ordination plot.

Two gradients are observed on the DCA simple ordination plot. The first gradient was related to the type of vegetation, going from quadrant I with open and low vegetation to quadrant III with high forest vegetation and large trees. Perpendicular to this gradient and thus independent of it, another gradient was observed ranging from riparian forests (quadrant II) to upland (no alluviality) in quadrant IV.

## Discussion

### An Interesting Method for Further Genetic Studies

Our study presents the first genetic analysis of wild populations of *Vitis* in North America in a sympatric region. Genetic analyses carried out thus far on North American species have been solely based on plants from collections.

i) The SSR markers used in this study were developed from repeat-rich genomic libraries made from nuclear DNA of *V. vinifera* and *V. riparia* cultivars (see **Table 1**), the ranges of length values of the PCR fragments were correct and showed polymorphism, ii) all the primers are regularly used on rootstocks and interspecific grape varieties (Lin and Walker, 1998; Upadhyay et al., 2007; Riaz et al., 2019; etc.), and iii) these primers are well distributed across the *Vitis* genome (Adam-Blondon et al., 2004), we decided to rely on these previous studies and did not sequence the amplified fragments (Arnold et al., 2002). For *Muscadinia* genus, this set of primers was not suitable and induced an artificial low genetic diversity. No *M. rotundifolia*-based SSR or EST-SSR markers were developed for this genus however other more suitable primer sets are available (Riaz et al., 2008). The development of other primers (Picq et al., 2014; Dong et al., 2018; etc.) linked to aspects of resistance, phenology or environmental adaptation may also be of interest for further population genetic analysis of local *Vitis*. The set of 23 SSR markers nevertheless seems appropriate for carrying out further genetic studies on local wild populations of *Vitis* in the United States and also on feral rootstock populations in Europe.

### Arkansas: A Sympatric Region for Genetically Diverse Vitaceae, Where Hybridization May Occur

Similar to many lowland forests of North America (Schnitzler et al., 2016), the forests of Arkansas are rich in Vitaceae, including several *Vitis sp,* as well as *M. rotundifolia* and *P. quinquefolia*. This situation does not occur in European forests where many plant species seem to have been eliminated during cold, dry glacial periods of the Pleistocene Era (2–0.001 Myr BP) (Adams and Woodward, 1989). Moreover, we have shown in our study that not only are *Vitis* abundant, but also that each species benefits from a high level of intraspecific genetic variability.

Even though *V. rupestris* was only present in two stations, genetic diversity was quite high. This plant is relatively discreet (personal observations, Pavek et al., 2003). It does not rise very high in the canopy, as shown by ecological analysis and botanical descriptions. It prefers open environments and is particularly adapted to alluvial sandy islands (Viala, 1889; Pavek et al., 2003). As early as (Munson, 1909) had noted its high ability to form natural hybrids, observed both in his collection and in nature in southwestern Missouri. He noted: *“Pollen of staminate plants very prepotent in fertilizing and hybridizing with other species.”* The fact that *V*. *rupestris* was found only in two stations does not mean that it is rare. It is of conservation concern in certain states like Kentucky, Pennsylvania, and Tennessee, due to land use practices of grazing in riparian areas and habitat loss from dams, spillways, and channelization (Moore, 1991; Pavek et al., 2003). While many causes of extinction have been mentioned for this species, it may also be necessary to take into account the presence of hybrids in these same alluvial zones that could accelerate the extinction of pure populations of *V. rupestris*. The high genetic diversity of *V. rupestris* along the Buffalo River suggests that this species is much more widespread than previously thought, in nearby valleys.

Hybrid and introgressed grapevines are rare in this region. Their pedigree indicates crosses between *V. riparia, V. aestivalis* and *V. rupestris*. We did not use chloroplast markers, but it would be interesting to see if these exchanges occurred in both directions. We were hoping to find hybrids with *V. berlandieri*, but this was not the case. *V. berlandieri* is a forest species with poor rooting ability. Natural hybrids including genes of *V. berlandieri* are perhaps less competitive in natural habitats. It might also be simply a matter of phenology. Indeed, in cultivation, plants may bloom at the same time but in a natural environment flowering periods are often not synchronized. This is, for example, the case in Europe with wild and cultivated grapevines. *V. vinifera* ssp *sylvestris* tends to bloom 2 weeks later than cultivars in vineyards (Arnold, 2002).

The only natural crossing between *V. aestivalis* and *V. rupestris* was found outside the alluvial zone. Crossings involving *V. riparia* and *V. aestivalis* were found on the first alluvial terrace, submitted to regular floods in contrast with the second terrace.

But interpretation of these results should nevertheless be made with caution. Hybridization seems to allow, for instance, *V. aestivalis* to enter alluvial areas and *V. rupestris* to escape from them. But the establishment success of these hybrids remains very low. This is probably due to competition with other plant species, but most likely to competition with their parents. We can assume that natural hybridization is in fact rather rare between the different *Vitis* species. This may be due to differences, for example, in phenology, viability of the seeds, etc. Moreover, viable *Vitis* crossings are perhaps repressed by their parents in the most suitable habitats of the region (Cheplick, 1992). We should also take into account that this landscape suffered from strong human impact for several centuries. The plateaus still show slow renaturation ongoing processes. Campbell et al. (2015) showed how lianas responded to forest fragmentation at the level of diversity and abundance. Further investigations should be made to link hybridization and anthropization.

The first natural crossing between these four *Vitis* species may have been complicated, but it is known that the interspecies hybrids and their progeny are fertile and vigorous. Once this first barrier has been crossed, their genetic diversity allows them to colonize new environments. This was shown in our study but it was also shown for other species such as *Centaurea stoebe* L. or *Prunus serotina* Ehrh. (Broennimann et al., 2007; Guisan et al., 2014). If parents (this also includes other Vitaceae such as *M. rotundifolia* and *P. quinquefolia*) are well established in the environment, crossings do not seem to spread, but if they arrive in a perturbed environment where their wild relative is weakened by biotic and abiotic pressures, they have the opportunity to develop rapidly. This is what we observed in Europe. In the valleys of tributaries of the Rhone in France, the hydrogeomorphology and geologic substrate are similar to those of the Buffalo River, and the ecological niche of *V. vinifera* ssp. *sylvestris* has been empty for decades. Interspecific natural crossings have therefore spread rapidly and intensively. In contrast, in the Danube alluvial forests of Austria where abiotic conditions are close to those of the Rhone tributaries, populations of wild grapevines are well-established and rootstock crossings do not enter the forest. Instead, they are established in anthropized zones along the main stream (Arnold et al., 2017).

### A Subtle Share of Space in Arkansas Forests

The forests of Arkansas display abundant supplies of water and nutrients during the growing season, which is highly favorable for ascending plants. Vitaceae, however, have similar strategies of climbing (i.e., tendrils and twining), which may enhance competition. Our study has nevertheless shown a subtle share of space at the local scale. *P. quinquefolia* seems to be the most opportunistic species, occupying all strata, and to have the largest range of habitats. Its strategy of adhesive tendrils limits the number of secondary ramets and may explain the larger sizes. It also shows efficient vegetative reproduction from hardwood cuttings or layering (USDA, 2019). It grows in most moist soil types but is resistant to drought. It grows well in full sun but also tolerates shade. Its seeds are particularly attractive to birds and have a high rate of germination success even in shade USDA, 2019). Such tools explain its abundance not only at the local scale (in both alluvial forests of the Buffalo River and adjacent forests), but also everywhere in the region, although mostly in eastern and midwestern United States (Coladoanto, 1991).

In contrast, *M. rotundifolia* occupies a small niche restricted to the first terraces of alluvial forests, in which it is present only in the canopy. This may be because seedling survival and establishment in understoreys is prevented by root and shoot competition with other Vitaceae.

In general, all four *Vitis* species are present throughout the Buffalo River region. But when we look to a smaller scale—and this is a strength of this study—we realize the subtle harmony that allows these species to coexist without any apparent competition.

Among *Vitis* species, we clearly see a hierarchy in the colonization of riverine forests of the Buffalo River. *V. riparia* is the most abundant and the most widespread of all. But compared with *P. quinquefolia,* which is also widespread along the river, young individuals of *V. riparia* are less abundant and limited to full sun sites.

The DCA analysis clearly shows that *V. rupestris* is linked to rather bushy vegetation and to alluvial islands made of sand. This corresponds to the literature (Viala, 1889; USDA, 2019). Nevertheless, this environment is highly unstable. Islands and riversides are frequently modified in shape and size during flooding. According to the literature, this species is particularly suitable for rooting and marcotting (USDA, 2019). As a result, when a sand deposit covers the plant, or when it is uprooted by flooding, it has a particularly high capacity to survive. The other species of *Vitis* are much less competitive in this environment.

The presence of *V. berlandieri* in alluvial areas is not common, according to the literature (Uretsky, 2018). For a long time, the consensus was that this species was associated with drought tolerance in rootstocks (Carbonneau, 1985; Christensen, 2003), yet this contradicts the experience of winegrowers. Padgett-Johnson et al. (2003) showed that *V. berlandieri* is among the least drought-tolerant *Vitis* species. *V. berlandieri* issued from collections has been mainly collected and studied in the Texas region. Uretsky (2018) noted that this species is present in dry sites showing signs of recent flooding. From our observations, this species indeed seems to be linked to upland areas, but also to alluvial areas.

*V. aestivalis* finds its optimal situation in well-drained sites (Morano and Walker, 1995; Heinitz et al., 2019). At least in this region, this species is exclusively located in the upland on the plateaus. It has high heat tolerance (Reisch and Pratt, 1996). Along the river, this species is probably limited by temporarily anoxic conditions. But according to the USDA PLANTS database (USDA, 2019), it has a relatively wide geographic distribution, mainly colonizing coarse textured soils that undergo drought conditions. In our study, it was most often found in the herbaceous strata. Although genetically more distant from other *Vitis* species (Wan et al., 2013; Ma et al., 2018; Klein et al., 2018), it is well present in natural crossings. *V. aestivalis* does not propagate easily through dormant cuttings, which is probably why it has historically been more important in breeding fruiting French-American varieties than in rootstock development (Keller, 2020). The Norton grape cultivar, for instance, is derived from *V. vinifera* and *V. aestivalis*. We can therefore say that, even though the aforementioned species appear to live in total harmony along the Buffalo River at a large scale, they share the space in a very specific way. They find their optimal growth in microenvironments within the same surfaces.

### Possible Scenarios for Europe

In Europe, previous studies have shown that *V. vinifera* ssp *sylvestris* was adapted to several different environments within its range before its near extinction in the 20th century (Arnold, 2002; Ocete et al., 2008; Ocete et al., 2011). It was present in colluvial zones made of large rocks, as well as in alluvial zones with silty soils. It was present in tall middle European forests, such as oak (several *Quercus* sp) or beech (*Fagus sylvatica* L.) forests, as well as in Mediterranean forests such as the “garrique.” In Iran, it grows in the temperate rain forests of the Alborz mountain range (Naqinezhad et al., 2018). Nowadays, this situation has gone forever, letting American crossings free to colonize habitats of the native species. Moreover, its invasive potential for living in a large range of habitats may be enhanced by the relative poverty in other native climbers (linked to paleogeographic reasons) even in the most favorable climatic parts of the Mediterranean basin (Schnitzler and Arnold, 2010).

Among American *Vitis* species, we think that *V. riparia* has the highest invasive potential. *V. riparia* is present from Canada to Texas and is abundant along French rivers and adjacent managed and natural ecosystems (personal observations in France, Germany, Austria, Italy, Spain). But hybridization might give potentialities for other *Vitis* species to adapt to new environments, as observed along the Buffalo River with crossings of *V. rupestris* and *V. riparia,* which are present on more erosive parts of alluvial zones, such as islands.

## Conclusion

This local study only shows trends, but it demonstrates the importance of paying attention at an early stage to the ecology and genetics of related crop species in their native natural environments. This type of local study should be conducted in several locations around the world where *Vitis* species are present. It is obvious that the behavior of plants in their natural environment and in cultivation is often very different. It is also necessary to take into account the geographic origin of germplasm to better understand not only its real potential in viticulture, but also the environmental dangers it can represent in areas of introduction. With human-induced cross-breeding, we bring an additional dimension to invasive risk. In this case, we are dealing with species that are capable of hybridizing and naturally introgressing in their area of origin. We see that they are controlled by biotic and abiotic conditions in healthy environments, but what can we expect from their behavior and evolution in an introductory region with no real competition from local parent species, in a general context of low competitivity with other lianas, and with a high degree of anthropization? Better knowledge of the ecology of these hybrid species in Europe is necessary. This knowledge must be built not only to better understand and possibly control the spread of these crossings in nature, but also to alert plant breeders of the need to perhaps consider this new emergence of ferality and the natural selection of hybrids as a great opportunity to find new germplasm available for the development of a more sustainable viticulture.

## Data Availability Statement

The datasets generated for this study are included in the article/**Supplementary File**.

## Author Contributions

CA did the field work, the genetic analysis, most statistics, and the co-writing. AS did the field work, some statistics, and co-writing.

## Funding

The field trip was supported by the Fondation Joachim de Giacomi of the Swiss Academy of Science (ScNAT).

## Conflict of Interest

The authors declare that the research was conducted in the absence of any commercial or financial relationships that could be construed as a potential conflict of interest.

## Supplementary Material

SM1 Three pictures showing the alluvial landscape during the flood where Vitaceae species were present (Buffalo River, Arkansas, United States).

SM2 Ecological data.

SM3 Genetic data.
