# Supplementary figures and images for: Ecology and Genetics of Natural Populations of North American Vitis Species Used as Rootstocks in European Grapevine Breeding Programs
Source: Front Plant Sci. 2020 Jun 19;11:866. doi: 10.3389/fpls.2020.00866 (PMC7319040; doi:10.3389/fpls.2020.00866)

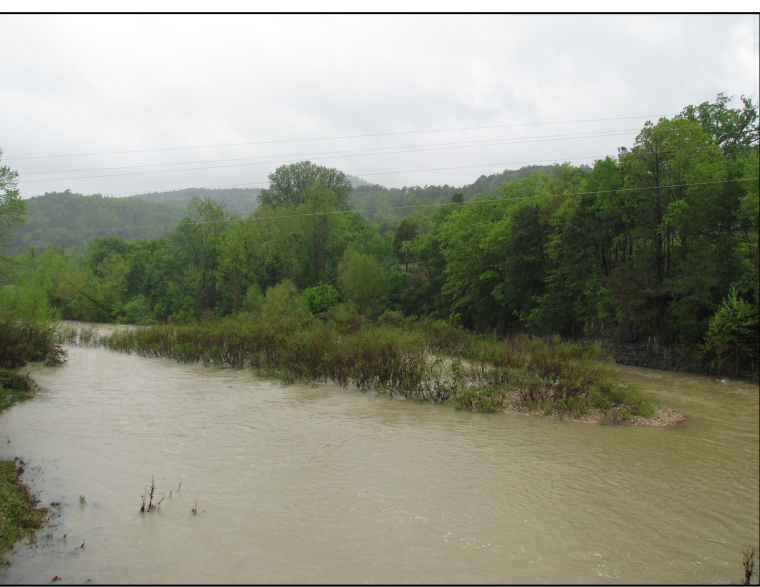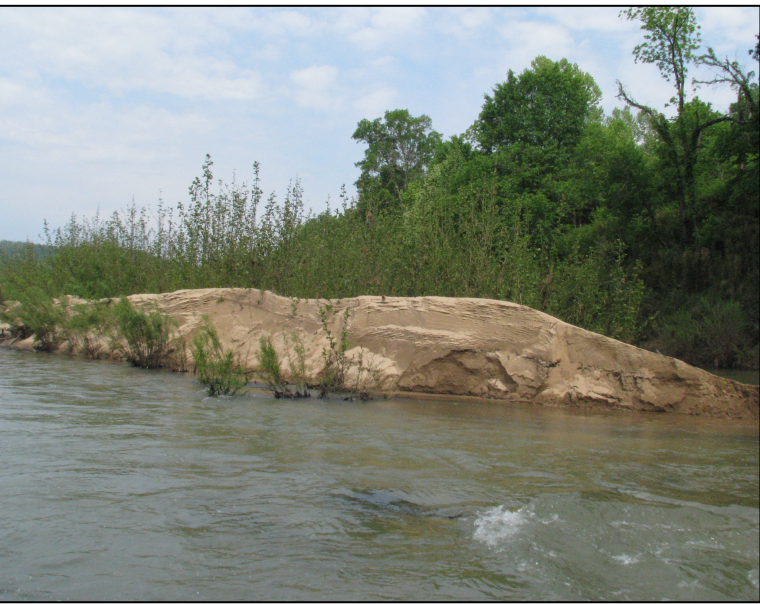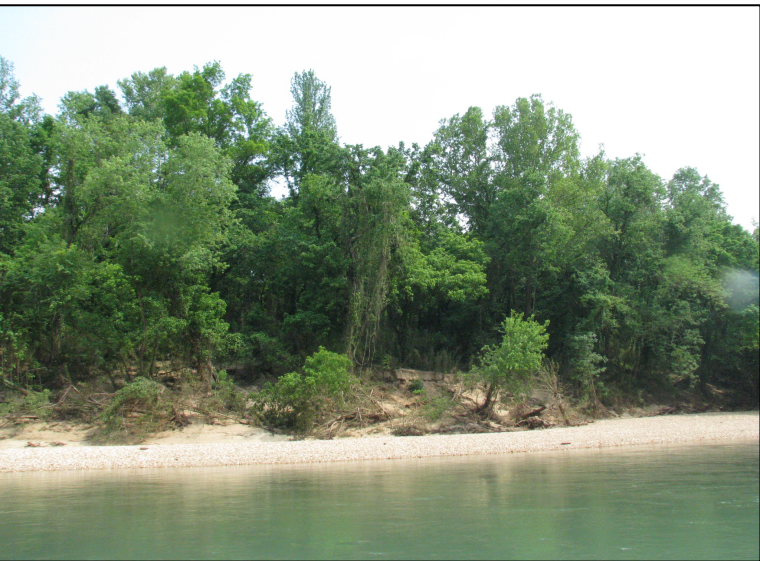

Supplement: SM1 [file DataSheet_1.pdf]
