# Supplementary material for: Ecology and Genetics of Natural Populations of North American Vitis Species Used as Rootstocks in European Grapevine Breeding Programs
Source: Front Plant Sci. 2020 Jun 19;11:866. doi: 10.3389/fpls.2020.00866 (PMC7319040; doi:10.3389/fpls.2020.00866)
Supplement: SM2 [file DataSheet_2.pdf]

	V. riparia	*V. hybrids*	*V. aestivalis*	*V. rupestris*	*V. berlandieri*	*M. rotundifolia*	Dip30	D1030	Dim10
St01	1						2	12	10
St02	1						3	6	20
St03			1			1	3	7	13
St04	1						1		4
St05	1						1	3	16
St06			1				3	9	17
St07		1	1				10	8	4
St08	1						1	8	3
St09	1					1	3	18	2
St10	1					1	4		
St11					1	1	2	5	2
St12	1				1			5	20
St13					1	1	6	10	7
St17	1				1	1	2	6	20
St18	1						5	9	4
St19	1						1	6	2
St20	1					1	8	6	3
St21	1						7	9	9
St22	1					1	5	14	11
St24					1	1		11	4
St26					1	1	5	4	
St29	1						4	18	22
St30	1					1	4	21	12
St33	1					1	14	7	14
St35	1						0	0	100
St36	1						3	5	20
St37	1						4	8	10
St38	1							5	10
St40	1					1		19	17
St41	1				1	1		2	50
St42	1	1				1			
St43	1					1	6	13	5
St44	1						4	4	7
St45	1	1			1	1	4	26	10
St46			1				1	16	
St47	1		1				3	8	24
St48	1						5	9	40
St49		1		1				11	20
St50	1	1					2	12	50
St51	1						4	15	19
St52	1	1				1	8	12	10
St53	1						4	11	20
St54			1				7	18	18
St55				1	1		1	10	18
St56			1				2	7	10
St57	1					1	7	7	5
St58					1	1		15	4
St59	1					1	6	12	9
St60					1	1	9	11	2

	V. riparia	*V. hybrids*	*V. aestivalis*	*V. rupestris*	*V. berlandieri*	*M. rotundifolia*	Dip30	D1030	Dim10
St61	1						7	6	27
St62	1					1	10	14	23
St63	1					1		13	6
St64	1						7	12	30
St66					1	1	1	15	21
St68					1		2	5	30
St69	1				1			11	25
St71					1	1	9	3	20
St72	1						5	7	20
St73	1					1	3	10	17
St74					1	1	2	10	2
St75					1		8	10	6

	Islnd	RipFo	Tce1	Tce2	NonAl	C.O.	CanoH	UndCa	NVitC	DmaVC
St01		1		1		20	20	10	9	8
St02		1		1		90	20	8	1	6
St03		1		1		10	25	10	9	13
St04		1		1		20	25	15	5	25
St05					1	80	10	5	6	4
St06					1	60	15	10	2	4
St07					1	30	18	12		
St08		1	1			50	20	10	15	8
St09		1	1			90	10	5	5	7
St10		1	1			100	15	15		
St11		1	1			30	25	12	6	8
St12		1		1		100	15	15	5	4
St13		1		1		40	25	25	4	8
St17		1		1		80	15	15	2	4
St18		1		1		30	25	18	7	15
St19		1	1			10	25	20	6	5
St20		1	1			40	25	15	5	7
St21		1	1			20	25	20	6	10
St22		1	1			40	25	20	12	10
St24		1	1			50	15	15	6	6
St26		1		1		60	25	20	4	10
St29					1	20	20	20	8	7
St30		1	1			30	15	15	9	5
St33		1		1		50	15	15	9	15
St35					1	100	3	2		
St36					1	40	18	18	2	9
St37					1	50	18	18	5	7
St38					1	90	8	8		
St40		1	1			20	10	10	10	4
St41	1	1	1			100	6	5		
St42	1	1	1			100	1	1		
St43		1	1			20	25	25	2	8
St44		1		1		30	25	25	5	10
St45		1	1			30	15	15	1	4
St46					1	20	18	18	14	3
St47	1	1	1			30	15	12	8	8
St48		1		1		40	25	15	5	5
St49		1	1			80	10	5	5	2
St50	1	1	1			80	15	15		
St51					1	20	20	10	2	4
St52		1	1			30	25	18	10	5
St53	1	1	1			60	25	10	3	2
St54		1		1		20	30	15	7	8
St55		1	1			70	8	5	2	7
St56		1	1			40	15	6	2	4
St57		1		1		60	8	8	5	7
St58		1		1		50	10	10	10	3
St59		1		1		30	25	20	2	5
St60		1		1		40	20	15	8	8

	Islnd	RipFo	Tce1	Tce2	NonAl	C.O.	CanoH	UndCa	NVitC	DmaVC
St61		1		1		60	20	6	3	5
St62		1	1			50	20	8	6	8
St63	1	1	1			70	15	15	3	5
St64		1		1		20	20	10	6	9
St66		1		1		20	20	15	7	12
St68		1		1		30	8	8	4	3
St69	1	1	1			90	7	2	4	2
St71		1		1		20	25	20	1	5
St72	1	1	1			80	10	8	1	2
St73		1		1		50	20	10	2	9
St74		1	1			60	10	10	4	4
St75		1		1		30	20	15	9	7

	NVitB	NMusC	DmaMC	NParC	DmaPC	NParB	PartH	VitiH
St01				5	3	2	2,1	0,5
St02				9	3	7	2,1	
St03		4	2	5	2		1	1,1
St04				6	3	2	1	
St05	10			8	1		2	2
St06	2						3	2
St07	2					1	4	3
St08				8	2	1	2	
St09	1	4	5	5	3		0,5	
St10	10	1	2	1	1	1	0,5	
St11		4	6					
St12	2			1	2	2	3	
St13		2	8	3	1	4	2	
St17	10	2	5	2	2	4	0,5	0,5
St18				13	5		2	
St19								
St20		7	10	10	4		0,5	
St21				11	4		1	
St22		3	10	12	4	3	2	
St24	7	3	5	4	2	1		
St26		4	8	1	1	1		
St29							0,5	
St30	1	7	6	5	3		0,5	
St33		7	14	8	5			
St35	50						0,5	
St36				2	1	1	0,5	
St37	1			5	2		2	
St38	5					3	2	1
St40		2	4	13	2	2	3	
St41	2	1	4					
St42	0,5	1	4					
St43	1	5	9	5	2			
St44				6	3	1	1	0,5
St45		10	5					
St46	2						1	1
St47	2					1	1	
St48	3						2	
St49	4						0,5	
St50	2						0,5	0,5
St51							1	0,5
St52		2	5	2	4	2		
St53				5	2		2	0,5
St54				10	4		1	
St55	10							
St56	10			1	3		1	
St57		2	4	13	3	4		
St58	1	1	4	5	2	7		
St59		7	9	5	3	5		
St60		2	8	16	2	3	2	

	NVitB	NMusC	DmaMC	NParC	DmaPC	NParB	PartH	VitiH
St61	1			9	2	1	2	
St62		3	6	10	1	5		
St63	4	5	5	3	1			
St64							0,5	
St66		2	5	3	1			
St68	2							
St69	1			3	1			
St71		8	6	12	4		1	
St72				1	1			
St73	1	1	4	12	3	5	3	
St74		2	6	7	2	6	0,5	
St75				6	2		1	
