# Supplementary material for: Ecology and Genetics of Natural Populations of North American Vitis Species Used as Rootstocks in European Grapevine Breeding Programs
Source: Front Plant Sci. 2020 Jun 19;11:866. doi: 10.3389/fpls.2020.00866 (PMC7319040; doi:10.3389/fpls.2020.00866)
Supplement: SM3 [file DataSheet_3.pdf]

Sample	VMC 2F10-1	VMC 2F10-2	VMC 5G8-1	VMC 5G8-2	VMC 6E10-1	VMC 6E10-2
St03-1_Ae	0	0	0	0	0	0
St06-1_Ae	83	110	0	0	91	93
St07-1_Ae-Ru	99	103	285	310	97	97
St46-3_Ae	103	105	291	301	93	103
St47-2_Ae	101	107	0	0	0	0
St54-1_Ae	103	103	301	303	97	99
St54-2_Ae	103	103	301	303	97	99
St56-1_Ae	103	105	299	303	95	103
St56-2_Ae	103	105	299	303	95	103
St11-1_Be	103	103	303	309	105	109
St12-2_Be	91	103	309	325	97	105
St13-1_Be	101	103	0	0	97	97
St17-2_Be	103	103	0	0	105	105
St24-1_Be-Ru	91	103	0	0	101	105
St26-1_Be	103	103	303	307	97	97
St26-2_Be	103	103	0	0	97	125
St41-1_Be	103	103	317	325	97	97
St45-2_Be	103	103	323	323	97	97
St55-1_Be	103	103	289	325	97	105
St58-1_Be	103	103	285	323	97	105
St60-1_Be	103	103	303	309	97	105
St66-1_Be	101	103	309	313	97	105
St68-1_Be	91	103	305	309	97	105
St69-1_Be	101	103	0	0	95	105
St71-1_Be	101	103	0	0	97	97
St74-1_Be	101	103	289	289	0	0
St75-1_Be	103	103	313	315	97	97
St03-2_MU	127	143	300	300	0	0
St10-2_MU	107	119	300	300	0	0
St17-3_MU	107	119	300	300	0	0
St20-2_MU	103	105	300	300	0	0
St22-2_MU	103	107	300	300	0	0
St26-3_MU	0	0	300	300	0	0
St33-2_MU	107	133	300	300	0	0
St40-2_MU	0	0	300	300	0	0
St41-3_MU	107	133	300	300	0	0
St43-1_MU	101	101	300	300	0	0
St45-3_MU	113	133	300	300	0	0
St52-3_MU	107	133	300	300	0	0
St57-1_MU	107	133	300	300	0	0
St58-2_MU	133	143	300	300	0	0
St59-2_MU	133	143	300	300	0	0
St60-2_MU	107	133	300	300	0	0
St62-2_MU	119	119	300	300	0	0
St66-2_MU	119	119	300	300	0	0
St71-2_MU	133	143	300	300	0	0
St43-2_Ri-CI	103	103	0	0	89	105
St01-1_Ri	103	103	305	305	95	99
St01-2_Ri	103	103	301	307	95	105
St02-1_Ri	103	103	300	305	95	99
St04-1_Ri	103	103	303	305	89	103
St05-1_Ri	103	103	298	301	99	101

Sample	VMC 2F10-1	VMC 2F10-2	VMC 5G8-1	VMC 5G8-2	VMC 6E10-1	VMC 6E10-2
St05-2_Ri	103	103	303	305	95	95
St08-1_Ri	103	103	301	305	95	99
St09-1_Ri	103	103	307	310	95	103
St10-1_Ri	103	103	305	305	101	115
St12-1_Ri	103	105	301	305	93	95
St17-1_Ri	103	103	301	305	91	95
St18-1_Ri	103	103	303	305	95	103
St20-1_Ri	103	105	287	303	99	111
St21-1_Ri	103	107	301	305	101	101
St22-1_Ri	103	103	301	302	105	111
St29-2_Ri	103	103	302	305	99	103
St29-3_Ri	103	103	301	301	99	101
St30-1_Ri	103	103	0	0	99	105
St33-1_Ri	103	103	0	0	99	99
St35-1_Ri	103	103	297	303	95	111
St36-2_Ri	83	103	307	307	99	99
St37-1_Ri	103	103	301	303	89	95
St37-2_Ri	103	109	301	303	93	93
St38-1_Ri	103	109	301	305	97	101
St38-2_Ri	103	109	300	302	95	107
St38-3_Ri	103	109	289	301	89	89
St40-1_Ri	103	105	303	303	89	89
St41-2_Ri	103	103	303	303	95	115
St42-1_Ri-Ae	103	103	289	305	89	95
St44-1_Ri	103	103	305	305	95	101
St45-1_Ri-Ae	103	103	0	0	95	103
St47-1_Ri	101	109	301	305	89	125
St48-1_Ri	103	103	303	305	99	99
St50-2_Ri-Ae	103	103	301	301	103	111
St51-1_Ri	103	103	303	303	93	97
St51-2_Ri	105	105	303	303	95	99
St51-3_Ri	103	103	301	302	99	99
St52-1_Ri	103	109	298	302	93	95
St52-2_Ri-Ae	103	103	303	305	93	99
St53-1_Ri	103	103	301	310	95	99
St57-2_Ri	103	103	303	305	95	95
St59-1_Ri	83	103	305	305	89	95
St61-1_Ri	103	103	301	310	103	111
St62-1_Ri	103	103	305	308	95	101
St63-1_Ri	103	103	305	311	99	101
St64-1_Ri	103	103	305	305	101	111
St69-2_Ri-Ru	103	103	301	305	89	101
St72-1_Ri-Ae	103	105	301	305	99	99
St73-1_Ri	103	103	298	300	95	115
St49-1_Ru	103	103	305	313	89	99
St49-2_Ru	103	103	305	313	89	99
St49-3_Ru-Ri	103	103	305	313	95	99
St55-2_Ru	85	87	301	305	89	101
St55-3_Ru	85	87	301	305	89	101

Sample	VMC 7F2-1	VMC 7F2-2	VMC 8G6-1	VMC 8G6-2	VMC 9B5-1	VMC 9B5-2
St03-1_Ae	0	0	0	0	0	0
St06-1_Ae	198	202	137	140	0	0
St07-1_Ae-Ru	196	200	135	137	234	237
St46-3_Ae	200	210	135	153	234	235
St47-2_Ae	0	0	143	143	0	0
St54-1_Ae	194	194	142	165	234	235
St54-2_Ae	194	194	142	165	234	235
St56-1_Ae	208	210	0	0	235	237
St56-2_Ae	208	210	135	137	235	237
St11-1_Be	194	202	140	140	229	237
St12-2_Be	194	194	135	140	228	233
St13-1_Be	194	202	140	140	0	0
St17-2_Be	194	202	133	140	252	252
St24-1_Be-Ru	194	194	0	0	0	0
St26-1_Be	194	204	0	0	233	252
St26-2_Be	194	202	133	133	229	240
St41-1_Be	194	194	133	140	233	240
St45-2_Be	194	208	133	133	237	252
St55-1_Be	204	204	0	0	237	241
St58-1_Be	194	208	133	133	229	252
St60-1_Be	194	194	0	0	252	252
St66-1_Be	204	204	0	0	228	230
St68-1_Be	194	208	0	0	237	237
St69-1_Be	194	194	0	0	250	250
St71-1_Be	194	194	0	0	233	252
St74-1_Be	194	194	0	0	237	237
St75-1_Be	194	202	137	140	229	229
St03-2_MU	218	220	0	0	222	222
St10-2_MU	218	220	127	127	222	222
St17-3_MU	218	220	127	127	222	222
St20-2_MU	0	0	0	0	222	222
St22-2_MU	218	220	0	0	222	222
St26-3_MU	220	222	127	127	222	222
St33-2_MU	218	220	0	0	222	222
St40-2_MU	0	0	0	0	222	222
St41-3_MU	218	220	127	127	222	222
St43-1_MU	218	220	127	127	222	222
St45-3_MU	218	220	127	127	222	222
St52-3_MU	222	222	127	127	222	222
St57-1_MU	222	222	127	127	222	222
St58-2_MU	0	0	0	0	222	222
St59-2_MU	218	220	127	127	220	222
St60-2_MU	218	220	0	0	222	222
St62-2_MU	222	222	127	127	222	222
St66-2_MU	218	222	127	127	222	222
St71-2_MU	218	220	127	127	222	222
St43-2_Ri-CI	194	194	0	0	235	237
St01-1_Ri	200	200	0	0	238	241
St01-2_Ri	194	218	135	137	229	235
St02-1_Ri	194	200	134	135	231	240
St04-1_Ri	194	202	135	135	231	235
St05-1_Ri	194	204	0	0	231	238

Sample	VMC 7F2-1	VMC 7F2-2	VMC 8G6-1	VMC 8G6-2	VMC 9B5-1	VMC 9B5-2
St05-2_Ri	194	194	0	0	233	235
St08-1_Ri	200	200	134	136	231	237
St09-1_Ri	194	194	135	137	237	237
St10-1_Ri	208	208	0	0	237	237
St12-1_Ri	200	202	135	137	235	238
St17-1_Ri	194	194	133	163	233	237
St18-1_Ri	194	202	0	0	237	237
St20-1_Ri	194	202	134	136	231	235
St21-1_Ri	194	194	134	135	231	233
St22-1_Ri	194	194	0	0	231	238
St29-2_Ri	194	208	135	149	233	238
St29-3_Ri	194	200	0	0	231	233
St30-1_Ri	194	200	134	165	235	237
St33-1_Ri	194	200	137	163	231	237
St35-1_Ri	200	200	133	161	231	231
St36-2_Ri	194	194	135	142	231	235
St37-1_Ri	194	204	135	135	238	245
St37-2_Ri	194	218	134	135	233	237
St38-1_Ri	194	196	135	135	238	243
St38-2_Ri	194	200	134	136	231	237
St38-3_Ri	194	196	134	136	231	237
St40-1_Ri	194	194	134	135	235	235
St41-2_Ri	194	206	135	135	231	233
St42-1_Ri-Ae	194	194	133	157	235	235
St44-1_Ri	194	194	134	161	231	231
St45-1_Ri-Ae	194	208	0	0	0	0
St47-1_Ri	194	200	134	149	235	236
St48-1_Ri	194	194	135	153	231	231
St50-2_Ri-Ae	200	202	142	155	237	242
St51-1_Ri	194	218	135	151	231	233
St51-2_Ri	194	194	0	0	231	233
St51-3_Ri	194	196	0	0	231	231
St52-1_Ri	194	200	134	134	233	237
St52-2_Ri-Ae	194	194	133	135	236	237
St53-1_Ri	194	194	134	136	230	231
St57-2_Ri	194	194	134	135	235	235
St59-1_Ri	194	194	0	0	237	237
St61-1_Ri	194	200	135	135	235	237
St62-1_Ri	194	200	132	137	231	235
St63-1_Ri	194	194	135	137	235	236
St64-1_Ri	208	218	134	135	231	235
St69-2_Ri-Ru	200	202	0	0	231	242
St72-1_Ri-Ae	194	200	134	137	233	235
St73-1_Ri	194	196	133	134	229	231
St49-1_Ru	200	202	0	0	237	238
St49-2_Ru	200	202	135	161	237	238
St49-3_Ru-Ri	194	202	135	136	237	237
St55-2_Ru	194	194	128	136	238	238
St55-3_Ru	194	194	0	0	238	238

Sample	ZAG 112-1	ZAG 112-2	ZAG 83-1	ZAG 83-2	VMC 1E8-1	VMC 1E8-2
St03-1_Ae	0	0	0	0	0	0
St06-1_Ae	246	248	0	0	0	0
St07-1_Ae-Ru	243	246	191	193	218	218
St46-3_Ae	245	246	191	192	212	214
St47-2_Ae	0	0	0	0	0	0
St54-1_Ae	233	251	191	191	0	0
St54-2_Ae	233	251	191	191	0	0
St56-1_Ae	249	251	191	191	208	218
St56-2_Ae	249	251	191	191	208	218
St11-1_Be	230	230	162	167	219	231
St12-2_Be	230	230	167	191	212	219
St13-1_Be	0	0	0	0	0	0
St17-2_Be	230	230	171	173	214	219
St24-1_Be-Ru	0	0	0	0	0	0
St26-1_Be	230	230	169	173	214	214
St26-2_Be	230	230	173	191	214	214
St41-1_Be	238	250	169	169	210	229
St45-2_Be	230	230	167	169	214	229
St55-1_Be	230	230	169	185	221	227
St58-1_Be	230	230	169	191	210	216
St60-1_Be	230	230	167	185	214	233
St66-1_Be	230	230	167	173	221	231
St68-1_Be	230	230	185	185	208	227
St69-1_Be	230	230	169	185	208	210
St71-1_Be	230	230	162	191	0	0
St74-1_Be	230	230	167	171	208	212
St75-1_Be	230	230	167	171	219	221
St03-2_MU	234	234	197	223	0	0
St10-2_MU	235	235	197	223	0	0
St17-3_MU	234	235	197	223	0	0
St20-2_MU	234	235	192	197	0	0
St22-2_MU	235	235	197	223	0	0
St26-3_MU	235	235	191	197	0	0
St33-2_MU	234	235	197	223	0	0
St40-2_MU	234	234	191	197	224	224
St41-3_MU	234	235	197	223	0	0
St43-1_MU	235	235	0	0	0	0
St45-3_MU	234	234	197	223	0	0
St52-3_MU	234	234	197	223	0	0
St57-1_MU	235	235	197	223	0	0
St58-2_MU	234	235	197	223	0	0
St59-2_MU	234	235	197	223	0	0
St60-2_MU	234	234	197	223	0	0
St62-2_MU	234	235	197	223	0	0
St66-2_MU	234	234	197	223	0	0
St71-2_MU	234	234	197	223	0	0
St43-2_Ri-CI	249	281	191	191	210	210
St01-1_Ri	257	287	191	191	223	223
St01-2_Ri	277	291	191	191	208	220
St02-1_Ri	243	287	191	191	223	223
St04-1_Ri	245	251	162	191	0	0
St05-1_Ri	257	275	191	191	218	231

Sample	ZAG 112-1	ZAG 112-2	ZAG 83-1	ZAG 83-2	VMC 1E8-1	VMC 1E8-2
St05-2_Ri	253	275	162	191	208	228
St08-1_Ri	245	265	191	191	208	208
St09-1_Ri	249	267	191	191	226	228
St10-1_Ri	255	275	191	191	214	214
St12-1_Ri	247	267	162	191	207	217
St17-1_Ri	245	247	191	191	0	0
St18-1_Ri	239	251	162	191	208	209
St20-1_Ri	243	273	191	191	220	0
St21-1_Ri	239	259	191	191	214	232
St22-1_Ri	235	235	191	197	220	238
St29-2_Ri	255	259	191	191	208	208
St29-3_Ri	235	239	162	191	213	237
St30-1_Ri	253	255	191	191	0	0
St33-1_Ri	237	245	191	197	208	220
St35-1_Ri	247	249	191	191	239	239
St36-2_Ri	239	241	191	191	0	0
St37-1_Ri	243	247	191	191	204	210
St37-2_Ri	241	243	162	191	216	229
St38-1_Ri	255	255	191	191	0	0
St38-2_Ri	255	261	162	191	218	220
St38-3_Ri	239	265	191	197	218	218
St40-1_Ri	235	247	191	191	208	231
St41-2_Ri	243	249	191	191	208	210
St42-1_Ri-Ae	251	257	191	191	212	212
St44-1_Ri	243	251	162	191	215	235
St45-1_Ri-Ae	249	257	189	191	0	0
St47-1_Ri	245	249	191	191	208	213
St48-1_Ri	239	259	162	191	0	0
St50-2_Ri-Ae	245	261	191	191	212	214
St51-1_Ri	235	265	191	191	210	210
St51-2_Ri	243	255	191	191	208	209
St51-3_Ri	235	235	191	191	237	239
St52-1_Ri	239	251	162	162	209	210
St52-2_Ri-Ae	245	247	191	191	212	213
St53-1_Ri	241	253	191	197	226	232
St57-2_Ri	245	273	191	191	214	223
St59-1_Ri	239	281	162	191	209	235
St61-1_Ri	243	247	162	191	210	220
St62-1_Ri	245	251	191	191	218	220
St63-1_Ri	247	249	191	191	210	210
St64-1_Ri	249	255	191	191	220	220
St69-2_Ri-Ru	247	249	191	191	208	208
St72-1_Ri-Ae	235	251	191	191	213	218
St73-1_Ri	237	261	191	191	0	0
St49-1_Ru	243	279	162	191	216	221
St49-2_Ru	243	279	162	191	216	221
St49-3_Ru-Ri	245	279	162	191	210	221
St55-2_Ru	243	249	186	218	212	212
St55-3_Ru	243	249	186	218	212	212

Sample	VMC 2A5-1	VMC 2A5-2	VMC 5A1-1	VMC 5A1-2	VMC 5C5-1	VMC 5C5-2
St03-1_Ae	0	0	165	167	0	0
St06-1_Ae	0	0	162	170	0	0
St07-1_Ae-Ru	170	176	171	171	125	129
St46-3_Ae	162	162	171	171	117	119
St47-2_Ae	0	0	174	174	0	0
St54-1_Ae	170	194	167	173	123	125
St54-2_Ae	170	194	167	173	123	125
St56-1_Ae	178	194	169	175	117	125
St56-2_Ae	178	194	169	175	117	125
St11-1_Be	176	178	171	171	121	121
St12-2_Be	176	178	168	171	117	121
St13-1_Be	0	0	168	171	0	0
St17-2_Be	178	178	167	173	117	121
St24-1_Be-Ru	0	0	171	171	0	0
St26-1_Be	0	0	171	173	117	121
St26-2_Be	0	0	169	173	117	121
St41-1_Be	178	178	169	171	117	121
St45-2_Be	178	178	173	175	117	121
St55-1_Be	176	178	168	171	117	121
St58-1_Be	178	178	169	169	117	121
St60-1_Be	174	178	171	171	121	121
St66-1_Be	176	178	150	175	121	121
St68-1_Be	178	178	171	183	117	121
St69-1_Be	0	0	167	168	117	121
St71-1_Be	0	0	168	171	121	121
St74-1_Be	178	178	167	169	117	121
St75-1_Be	178	178	171	175	121	121
St03-2_MU	161	161	170	170	121	121
St10-2_MU	161	161	170	170	133	133
St17-3_MU	161	163	170	170	125	125
St20-2_MU	161	161	170	170	0	0
St22-2_MU	161	163	170	170	0	0
St26-3_MU	161	161	170	170	117	125
St33-2_MU	161	161	170	170	0	0
St40-2_MU	161	161	170	170	125	133
St41-3_MU	161	161	170	170	133	133
St43-1_MU	161	161	170	170	133	133
St45-3_MU	161	161	170	170	0	0
St52-3_MU	161	167	170	170	0	0
St57-1_MU	161	163	170	170	0	0
St58-2_MU	161	163	170	170	0	0
St59-2_MU	161	163	170	170	0	0
St60-2_MU	161	161	170	170	0	0
St62-2_MU	161	163	170	170	121	121
St66-2_MU	161	163	170	170	121	121
St71-2_MU	161	161	170	170	121	121
St43-2_Ri-CI	0	0	171	173	117	117
St01-1_Ri	166	194	179	181	125	133
St01-2_Ri	162	168	163	171	123	133
St02-1_Ri	176	194	160	181	123	125
St04-1_Ri	164	164	173	177	125	129
St05-1_Ri	172	184	167	177	117	125

Sample	VMC 2A5-1	VMC 2A5-2	VMC 5A1-1	VMC 5A1-2	VMC 5C5-1	VMC 5C5-2
St05-2_Ri	162	178	171	171	117	125
St08-1_Ri	164	198	169	169	117	125
St09-1_Ri	170	178	169	181	117	129
St10-1_Ri	178	180	169	169	117	129
St12-1_Ri	162	162	171	177	125	129
St17-1_Ri	0	0	163	179	125	127
St18-1_Ri	168	176	169	171	117	123
St20-1_Ri	168	172	169	169	117	125
St21-1_Ri	162	170	160	171	117	127
St22-1_Ri	0	0	171	171	117	125
St29-2_Ri	194	194	168	173	125	127
St29-3_Ri	170	184	167	169	125	133
St30-1_Ri	0	0	171	174	111	133
St33-1_Ri	196	196	156	169	115	117
St35-1_Ri	166	170	169	173	111	129
St36-2_Ri	170	195	169	179	125	125
St37-1_Ri	176	181	169	169	121	125
St37-2_Ri	162	168	167	171	125	127
St38-1_Ri	158	158	177	179	117	125
St38-2_Ri	176	178	165	167	121	125
St38-3_Ri	172	196	169	177	117	117
St40-1_Ri	170	176	169	171	125	125
St41-2_Ri	176	180	160	171	117	127
St42-1_Ri-Ae	162	170	169	169	0	0
St44-1_Ri	168	168	169	173	125	125
St45-1_Ri-Ae	0	0	169	169	0	0
St47-1_Ri	168	176	161	165	125	127
St48-1_Ri	174	180	160	171	111	117
St50-2_Ri-Ae	164	164	161	171	117	127
St51-1_Ri	178	180	169	169	123	125
St51-2_Ri	156	156	169	179	117	125
St51-3_Ri	168	186	177	181	111	135
St52-1_Ri	168	170	164	177	117	133
St52-2_Ri-Ae	168	174	169	175	125	133
St53-1_Ri	172	192	169	171	111	127
St57-2_Ri	168	196	169	171	125	129
St59-1_Ri	168	168	166	171	117	127
St61-1_Ri	178	198	171	179	125	125
St62-1_Ri	166	194	169	171	121	125
St63-1_Ri	168	176	171	171	125	125
St64-1_Ri	166	194	167	177	117	127
St69-2_Ri-Ru	166	174	171	179	125	129
St72-1_Ri-Ae	168	168	171	177	119	125
St73-1_Ri	168	192	163	171	117	125
St49-1_Ru	156	178	171	171	111	123
St49-2_Ru	156	178	171	171	111	123
St49-3_Ru-Ri	170	178	169	169	111	121
St55-2_Ru	166	174	161	161	121	127
St55-3_Ru	166	174	161	161	121	127

Sample	VVMD 24-1	VVMD 24-2	VVMD 25-1	VVMD 25-2	VVMD 31-1	VVMD 31-2
St03-1_Ae	213	213	0	0	200	200
St06-1_Ae	200	204	233	237	0	0
St07-1_Ae-Ru	200	204	247	253	0	0
St46-3_Ae	210	210	235	267	195	204
St47-2_Ae	0	0	259	259	208	208
St54-1_Ae	200	208	241	259	204	204
St54-2_Ae	200	208	241	259	204	204
St56-1_Ae	198	200	233	245	199	208
St56-2_Ae	198	200	233	245	199	199
St11-1_Be	200	202	255	255	204	204
St12-2_Be	200	202	249	255	204	204
St13-1_Be	202	206	0	0	0	0
St17-2_Be	200	204	263	265	202	204
St24-1_Be-Ru	204	204	0	0	202	202
St26-1_Be	202	202	251	251	202	204
St26-2_Be	200	202	249	251	204	206
St41-1_Be	200	202	249	251	202	204
St45-2_Be	200	202	249	263	202	204
St55-1_Be	206	206	241	249	202	204
St58-1_Be	200	202	241	263	202	204
St60-1_Be	200	202	241	265	204	204
St66-1_Be	200	206	251	263	202	202
St68-1_Be	200	200	241	251	204	208
St69-1_Be	202	206	247	249	204	206
St71-1_Be	202	202	257	259	204	204
St74-1_Be	200	200	245	257	199	199
St75-1_Be	200	200	241	255	204	204
St03-2_MU	227	227	0	0	199	199
St10-2_MU	227	227	241	251	199	199
St17-3_MU	227	227	243	257	199	199
St20-2_MU	227	227	0	0	0	0
St22-2_MU	227	227	0	0	199	220
St26-3_MU	227	227	0	0	0	0
St33-2_MU	227	227	0	0	199	221
St40-2_MU	227	227	0	0	197	204
St41-3_MU	227	227	0	0	0	0
St43-1_MU	227	227	0	0	199	199
St45-3_MU	227	227	0	0	0	0
St52-3_MU	227	227	0	0	199	199
St57-1_MU	227	227	0	0	0	0
St58-2_MU	227	227	0	0	0	0
St59-2_MU	227	227	0	0	0	0
St60-2_MU	227	227	0	0	199	199
St62-2_MU	227	227	0	0	199	199
St66-2_MU	227	227	0	0	0	0
St71-2_MU	227	227	0	0	199	199
St43-2_Ri-CI	200	202	251	259	204	204
St01-1_Ri	208	214	235	251	0	0
St01-2_Ri	200	200	247	247	192	229
St02-1_Ri	208	214	247	251	192	192
St04-1_Ri	200	206	251	259	220	220
St05-1_Ri	200	200	245	261	202	202

Sample	VVMD 24-1	VVMD 24-2	VVMD 25-1	VVMD 25-2	VVMD 31-1	VVMD 31-2
St05-2_Ri	200	200	251	261	206	208
St08-1_Ri	200	206	233	261	204	204
St09-1_Ri	200	210	0	0	218	218
St10-1_Ri	200	210	243	265	204	204
St12-1_Ri	202	206	239	245	0	0
St17-1_Ri	200	216	235	257	206	206
St18-1_Ri	200	204	237	253	204	204
St20-1_Ri	200	202	247	253	218	218
St21-1_Ri	202	210	239	241	215	215
St22-1_Ri	200	208	239	247	204	218
St29-2_Ri	200	204	0	0	204	204
St29-3_Ri	210	216	251	261	206	206
St30-1_Ri	195	208	0	0	0	0
St33-1_Ri	200	206	247	247	225	225
St35-1_Ri	200	208	237	259	223	223
St36-2_Ri	195	216	235	237	220	220
St37-1_Ri	208	214	257	265	192	192
St37-2_Ri	214	216	239	247	206	206
St38-1_Ri	195	202	239	251	218	218
St38-2_Ri	200	214	239	259	202	202
St38-3_Ri	200	210	235	251	220	220
St40-1_Ri	214	216	235	249	204	204
St41-2_Ri	216	227	247	261	210	220
St42-1_Ri-Ae	200	214	261	263	210	210
St44-1_Ri	200	200	247	259	204	204
St45-1_Ri-Ae	200	200	251	251	0	0
St47-1_Ri	200	216	243	265	218	218
St48-1_Ri	216	216	249	261	204	204
St50-2_Ri-Ae	198	216	245	247	212	216
St51-1_Ri	200	227	247	265	204	204
St51-2_Ri	206	222	242	249	204	206
St51-3_Ri	200	216	235	243	218	218
St52-1_Ri	200	216	235	253	204	204
St52-2_Ri-Ae	200	200	249	271	202	202
St53-1_Ri	200	200	247	257	199	212
St57-2_Ri	208	216	243	259	204	204
St59-1_Ri	202	218	255	257	218	218
St61-1_Ri	200	216	235	251	204	204
St62-1_Ri	202	216	243	251	192	192
St63-1_Ri	195	200	245	247	210	220
St64-1_Ri	195	216	233	239	192	192
St69-2_Ri-Ru	200	200	247	247	204	204
St72-1_Ri-Ae	200	216	249	255	0	0
St73-1_Ri	200	210	255	259	192	199
St49-1_Ru	200	200	247	253	204	218
St49-2_Ru	200	200	247	253	204	218
St49-3_Ru-Ri	200	224	253	255	218	218
St55-2_Ru	202	204	235	235	200	202
St55-3_Ru	202	204	235	235	200	202

Sample	VVMD 32-1	VVMD 32-2	VMC 2B11-1	VMC 2B11-2	VMC 3D12-1	VMC 3D12-2
St03-1_Ae	261	261	0	0	0	0
St06-1_Ae	0	0	177	177	0	0
St07-1_Ae-Ru	0	0	177	180	208	210
St46-3_Ae	240	243	177	180	210	218
St47-2_Ae	240	240	0	0	0	0
St54-1_Ae	240	241	180	180	210	220
St54-2_Ae	240	241	180	180	210	220
St56-1_Ae	231	231	180	180	212	232
St56-2_Ae	231	231	180	180	212	232
St11-1_Be	236	258	177	177	205	208
St12-2_Be	248	250	177	177	209	209
St13-1_Be	0	0	177	177	0	0
St17-2_Be	268	270	177	177	208	209
St24-1_Be-Ru	256	260	0	0	0	0
St26-1_Be	244	266	177	177	0	0
St26-2_Be	244	248	177	177	0	0
St41-1_Be	236	248	177	177	208	208
St45-2_Be	236	248	177	177	209	209
St55-1_Be	250	270	177	180	209	210
St58-1_Be	236	248	177	177	208	211
St60-1_Be	238	244	177	177	0	0
St66-1_Be	248	248	177	177	209	209
St68-1_Be	248	260	177	177	208	208
St69-1_Be	254	266	177	177	0	0
St71-1_Be	248	250	0	0	0	0
St74-1_Be	0	0	177	177	209	209
St75-1_Be	250	268	177	177	208	209
St03-2_MU	0	0	171	171	0	0
St10-2_MU	235	241	171	171	0	0
St17-3_MU	0	0	171	171	0	0
St20-2_MU	0	0	171	171	0	0
St22-2_MU	0	0	171	171	0	0
St26-3_MU	251	251	171	171	0	0
St33-2_MU	245	251	171	171	0	0
St40-2_MU	0	0	171	171	0	0
St41-3_MU	0	0	171	171	210	214
St43-1_MU	235	245	171	171	205	210
St45-3_MU	0	0	171	171	0	0
St52-3_MU	0	0	0	0	0	0
St57-1_MU	0	0	171	171	0	0
St58-2_MU	0	0	171	171	0	0
St59-2_MU	0	0	171	171	0	0
St60-2_MU	0	0	171	171	0	0
St62-2_MU	0	0	171	171	0	0
St66-2_MU	0	0	171	171	0	0
St71-2_MU	234	234	171	171	0	0
St43-2_Ri-CI	239	239	180	180	0	0
St01-1_Ri	247	247	180	180	204	212
St01-2_Ri	241	241	177	180	210	214
St02-1_Ri	247	247	180	180	212	212
St04-1_Ri	239	239	180	183	210	218
St05-1_Ri	241	241	177	180	208	214

Sample	VVMD 32-1	VVMD 32-2	VMC 2B11-1	VMC 2B11-2	VMC 3D12-1	VMC 3D12-2
St05-2_Ri	249	249	180	183	210	222
St08-1_Ri	251	251	180	183	210	234
St09-1_Ri	237	244	174	180	210	210
St10-1_Ri	241	241	180	183	220	223
St12-1_Ri	239	239	177	180	210	212
St17-1_Ri	244	244	180	180	204	204
St18-1_Ri	245	255	180	183	210	234
St20-1_Ri	241	292	180	186	210	210
St21-1_Ri	239	239	174	180	210	212
St22-1_Ri	243	288	180	180	0	0
St29-2_Ri	0	0	177	180	210	212
St29-3_Ri	244	266	180	180	210	212
St30-1_Ri	243	246	177	180	0	0
St33-1_Ri	249	249	174	180	0	0
St35-1_Ri	241	250	180	180	210	210
St36-2_Ri	253	253	180	183	210	212
St37-1_Ri	243	255	180	180	210	212
St37-2_Ri	243	249	171	183	210	212
St38-1_Ri	245	245	180	180	210	220
St38-2_Ri	241	241	180	180	212	214
St38-3_Ri	241	241	180	180	212	212
St40-1_Ri	237	243	180	180	210	210
St41-2_Ri	237	241	174	180	210	210
St42-1_Ri-Ae	240	243	180	180	204	212
St44-1_Ri	243	243	180	183	212	212
St45-1_Ri-Ae	0	0	0	0	0	0
St47-1_Ri	247	247	183	183	210	212
St48-1_Ri	243	243	180	180	210	212
St50-2_Ri-Ae	0	0	174	180	210	223
St51-1_Ri	243	243	177	180	210	212
St51-2_Ri	255	255	180	180	214	218
St51-3_Ri	247	247	180	180	210	210
St52-1_Ri	247	247	174	180	210	212
St52-2_Ri-Ae	240	240	180	180	210	218
St53-1_Ri	241	241	180	183	210	210
St57-2_Ri	247	247	174	177	212	212
St59-1_Ri	243	243	180	183	208	210
St61-1_Ri	247	247	180	183	204	210
St62-1_Ri	241	244	177	180	204	218
St63-1_Ri	247	251	180	180	210	212
St64-1_Ri	242	246	0	0	210	232
St69-2_Ri-Ru	233	243	180	180	212	212
St72-1_Ri-Ae	253	253	180	186	208	210
St73-1_Ri	244	288	174	180	210	212
St49-1_Ru	241	241	180	180	200	212
St49-2_Ru	241	241	180	180	200	212
St49-3_Ru-Ri	241	292	180	180	208	212
St55-2_Ru	233	235	171	183	202	202
St55-3_Ru	233	235	171	183	202	202

Sample	VMC 4G6-1	VMC 4G6-2	VVMD 7-1	VVMD 7-2	VVS 2-1	VVS 2-2
St03-1_Ae	0	0	235	241	0	0
St06-1_Ae	0	0	235	235	141	145
St07-1_Ae-Ru	0	0	235	239	141	149
St46-3_Ae	0	0	237	239	123	151
St47-2_Ae	0	0	0	0	0	0
St54-1_Ae	119	119	237	237	137	139
St54-2_Ae	119	119	237	237	137	139
St56-1_Ae	123	123	237	239	139	141
St56-2_Ae	123	123	237	239	139	141
St11-1_Be	121	121	229	229	141	141
St12-2_Be	127	135	229	229	129	141
St13-1_Be	0	0	229	229	129	141
St17-2_Be	125	125	229	229	129	173
St24-1_Be-Ru	0	0	0	0	145	175
St26-1_Be	121	121	229	229	129	137
St26-2_Be	123	123	229	229	131	167
St41-1_Be	123	123	229	229	129	159
St45-2_Be	133	133	229	229	141	155
St55-1_Be	125	125	0	0	145	153
St58-1_Be	125	125	229	229	129	131
St60-1_Be	0	0	229	229	135	145
St66-1_Be	121	123	229	229	167	167
St68-1_Be	121	121	229	229	129	135
St69-1_Be	0	0	229	229	153	167
St71-1_Be	0	0	0	0	153	167
St74-1_Be	123	125	229	229	145	167
St75-1_Be	121	137	229	229	145	167
St03-2_MU	111	111	0	0	0	0
St10-2_MU	111	111	225	225	0	0
St17-3_MU	111	111	0	0	0	0
St20-2_MU	111	111	0	0	0	0
St22-2_MU	111	111	0	0	0	0
St26-3_MU	111	111	225	239	0	0
St33-2_MU	111	111	245	245	0	0
St40-2_MU	111	111	239	239	0	0
St41-3_MU	111	111	0	0	0	0
St43-1_MU	111	111	225	235	0	0
St45-3_MU	111	111	229	239	0	0
St52-3_MU	111	111	0	0	0	0
St57-1_MU	111	111	0	0	0	0
St58-2_MU	111	111	233	237	0	0
St59-2_MU	111	111	0	0	0	0
St60-2_MU	111	111	0	0	0	0
St62-2_MU	111	111	0	0	0	0
St66-2_MU	111	111	0	0	0	0
St71-2_MU	111	111	0	0	0	0
St43-2_Ri-CI	0	0	0	0	129	139
St01-1_Ri	119	119	239	239	137	137
St01-2_Ri	123	129	235	237	131	137
St02-1_Ri	119	119	239	239	131	137
St04-1_Ri	127	129	237	239	147	147
St05-1_Ri	0	0	239	239	133	145

Sample	VMC 4G6-1	VMC 4G6-2	VVMD 7-1	VVMD 7-2	VVS 2-1	VVS 2-2
St05-2_Ri	0	0	235	239	137	143
St08-1_Ri	115	139	239	239	125	139
St09-1_Ri	125	129	235	237	137	151
St10-1_Ri	0	0	239	239	139	149
St12-1_Ri	133	135	239	239	139	145
St17-1_Ri	129	131	0	0	133	137
St18-1_Ri	127	127	239	239	139	145
St20-1_Ri	115	131	225	239	135	141
St21-1_Ri	127	129	239	239	125	145
St22-1_Ri	0	0	227	239	137	145
St29-2_Ri	0	0	239	239	133	137
St29-3_Ri	119	125	239	239	137	145
St30-1_Ri	0	0	0	0	137	155
St33-1_Ri	123	131	0	0	137	153
St35-1_Ri	0	0	237	239	129	133
St36-2_Ri	0	0	239	239	125	145
St37-1_Ri	121	121	239	239	125	125
St37-2_Ri	119	121	239	239	126	137
St38-1_Ri	125	129	237	239	133	135
St38-2_Ri	131	133	237	239	137	139
St38-3_Ri	127	129	239	239	135	147
St40-1_Ri	0	0	239	239	135	153
St41-2_Ri	131	131	239	239	141	165
St42-1_Ri-Ae	0	0	225	239	135	139
St44-1_Ri	129	131	237	239	139	157
St45-1_Ri-Ae	0	0	0	0	141	145
St47-1_Ri	125	131	237	239	137	145
St48-1_Ri	123	129	239	245	137	163
St50-2_Ri-Ae	127	127	235	239	127	143
St51-1_Ri	127	131	239	241	131	139
St51-2_Ri	0	0	239	239	133	133
St51-3_Ri	0	0	239	239	137	143
St52-1_Ri	125	133	239	239	139	139
St52-2_Ri-Ae	119	125	239	241	143	143
St53-1_Ri	125	131	239	239	125	155
St57-2_Ri	119	129	235	239	125	131
St59-1_Ri	119	125	237	239	135	165
St61-1_Ri	121	135	237	239	135	159
St62-1_Ri	129	143	237	239	137	147
St63-1_Ri	131	135	239	239	125	135
St64-1_Ri	125	141	239	239	135	137
St69-2_Ri-Ru	0	0	239	239	143	163
St72-1_Ri-Ae	119	131	241	241	161	167
St73-1_Ri	0	0	239	239	133	135
St49-1_Ru	119	121	239	239	133	161
St49-2_Ru	119	121	239	239	133	161
St49-3_Ru-Ri	119	129	237	239	133	139
St55-2_Ru	125	127	249	249	139	141
St55-3_Ru	125	127	249	249	139	141

Sample	ZAG 62-1	ZAG 62-2	ZAG 79-1	ZAG 79-2
St03-1_Ae	203	209	291	297
St06-1_Ae	0	0	243	265
St07-1_Ae-Ru	179	213	255	257
St46-3_Ae	199	207	245	249
St47-2_Ae	174	177	0	0
St54-1_Ae	189	199	255	257
St54-2_Ae	189	199	255	257
St56-1_Ae	189	207	249	265
St56-2_Ae	189	207	249	265
St11-1_Be	174	215	245	261
St12-2_Be	174	215	251	261
St13-1_Be	0	0	245	251
St17-2_Be	178	187	249	251
St24-1_Be-Ru	0	0	0	0
St26-1_Be	174	174	255	259
St26-2_Be	0	0	251	251
St41-1_Be	174	174	251	267
St45-2_Be	195	207	251	267
St55-1_Be	174	174	0	0
St58-1_Be	195	215	245	257
St60-1_Be	187	203	251	251
St66-1_Be	187	187	255	257
St68-1_Be	174	174	245	245
St69-1_Be	178	178	245	255
St71-1_Be	0	0	245	255
St74-1_Be	174	174	251	255
St75-1_Be	174	215	245	251
St03-2_MU	0	0	0	0
St10-2_MU	181	198	227	227
St17-3_MU	165	196	227	227
St20-2_MU	196	198	0	0
St22-2_MU	181	196	227	227
St26-3_MU	181	196	0	0
St33-2_MU	181	194	227	227
St40-2_MU	194	198	255	255
St41-3_MU	181	194	227	227
St43-1_MU	194	194	227	227
St45-3_MU	181	194	227	227
St52-3_MU	181	181	227	227
St57-1_MU	196	200	227	227
St58-2_MU	181	196	253	259
St59-2_MU	194	196	0	0
St60-2_MU	181	196	259	259
St62-2_MU	181	196	253	259
St66-2_MU	165	165	227	227
St71-2_MU	165	165	227	227
St43-2_Ri-CI	193	195	251	251
St01-1_Ri	189	193	253	255
St01-2_Ri	193	195	249	261
St02-1_Ri	189	207	253	261
St04-1_Ri	193	213	249	263
St05-1_Ri	193	193	253	261

Sample	ZAG 62-1	ZAG 62-2	ZAG 79-1	ZAG 79-2
St05-2_Ri	207	207	241	259
St08-1_Ri	189	203	255	255
St09-1_Ri	193	213	241	259
St10-1_Ri	193	193	255	259
St12-1_Ri	193	195	253	265
St17-1_Ri	193	193	259	263
St18-1_Ri	189	193	257	263
St20-1_Ri	179	189	241	253
St21-1_Ri	179	193	241	249
St22-1_Ri	185	189	241	253
St29-2_Ri	191	191	253	263
St29-3_Ri	189	193	255	267
St30-1_Ri	193	193	259	261
St33-1_Ri	203	209	241	255
St35-1_Ri	193	207	251	253
St36-2_Ri	193	195	253	263
St37-1_Ri	193	211	241	255
St37-2_Ri	191	191	247	269
St38-1_Ri	207	219	253	261
St38-2_Ri	191	193	247	255
St38-3_Ri	197	207	257	261
St40-1_Ri	191	193	255	269
St41-2_Ri	185	189	255	255
St42-1_Ri-Ae	185	199	251	255
St44-1_Ri	185	195	253	257
St45-1_Ri-Ae	193	209	0	0
St47-1_Ri	193	207	253	263
St48-1_Ri	195	219	247	259
St50-2_Ri-Ae	193	207	247	251
St51-1_Ri	193	197	253	261
St51-2_Ri	189	191	241	249
St51-3_Ri	207	217	255	255
St52-1_Ri	203	209	255	269
St52-2_Ri-Ae	195	195	259	271
St53-1_Ri	193	207	251	255
St57-2_Ri	193	195	241	263
St59-1_Ri	195	197	255	267
St61-1_Ri	193	207	265	273
St62-1_Ri	193	207	249	259
St63-1_Ri	179	193	253	259
St64-1_Ri	179	193	255	257
St69-2_Ri-Ru	189	193	253	253
St72-1_Ri-Ae	195	195	249	257
St73-1_Ri	189	191	253	265
St49-1_Ru	179	213	249	261
St49-2_Ru	179	213	249	261
St49-3_Ru-Ri	195	213	241	249
St55-2_Ru	189	189	255	261
St55-3_Ru	189	189	255	261
